# DLGAP5 enhances bladder cancer chemoresistance by regulating glycolysis through MYC stabilization

**DOI:** 10.7150/thno.102730

**Published:** 2025-01-20

**Authors:** Zhao Deng, Fenfang Zhou, Mingxing Li, Wan Jin, Jingtian Yu, Gang Wang, Kaiyu Qian, Lingao Ju, Yi Zhang, Yu Xiao, Xinghuan Wang

**Affiliations:** 1 Department of Urology, Zhongnan Hospital of Wuhan University, Wuhan, China.; 2 Hubei Key Laboratory of Urological Diseases, Zhongnan Hospital of Wuhan University, Wuhan, China.; 3 Department of Radiology, Zhongnan Hospital of Wuhan University, Wuhan, China.; 4 Department of Biological Repositories, Human Genetic Resource Preservation Center of Hubei Province, Zhongnan Hospital of Wuhan University, Wuhan, China.; 5 Euler Technology, ZGC Life Sciences Park, Beijing, China.; 6 Center for Quantitative Biology, School of Life Sciences, Peking University, Beijing, China.; 7 Wuhan Research Center for Infectious Diseases and Cancer, Chinese Academy of Medical Sciences, Wuhan, China.; 8 Medical Research Institute, Frontier Science Center for Immunology and Metabolism, Taikang Center for Life and Medical Sciences, Wuhan University, Wuhan, China.

**Keywords:** DLGAP5, MYC, chemoresistance, glycolysis, bladder cancer

## Abstract

**Rationale:** Bladder cancer (BLCA), one of the most lethal urological tumors, exhibits high rates of recurrence and chemoresistance, particularly to gemcitabine (GEM). Understanding the mechanisms of GEM resistance is crucial for improving therapeutic outcomes. Our study investigates the role of DLGAP5 in promoting GEM resistance through modulation of glycolysis and MYC protein stability in BLCA cells.

**Methods:** We utilized various BLCA cell lines and clinical tissue samples to analyze the impact of DLGAP5 on GEM resistance. Through biochemical assays, protein interaction studies, and gene expression analyses, we examined how DLGAP5 interacts with USP11 and MYC, assessed the effects on MYC deubiquitination and stability. The influence of these interactions on glycolytic activity and GEM resistance was also evaluated via mouse subcutaneous xenograft model and spontaneous BLCA model.

**Results:** Our findings indicate that DLGAP5 enhances GEM resistance by stabilizing MYC protein via deubiquitination, a process mediated by USP11. DLGAP5 was found to facilitate the interaction between USP11 and MYC, promoting MYC-driven transcription of DLGAP5 itself, thereby creating a positive feedback loop. This loop leads to sustained MYC accumulation and increased glycolytic activity, contributing to GEM resistance in BLCA cells.

**Conclusion:** The study highlights the critical role of DLGAP5 in regulating MYC protein stability and suggests that disrupting the DLGAP5-USP11-MYC axis may provide a novel therapeutic approach to overcome GEM resistance in BLCA. DLGAP5 represents a potential target for therapeutic intervention aimed at mitigating chemoresistance in bladder cancer BLCA.

## Introduction

Bladder cancer (BLCA) originates from epithelial cells of the bladder and is a highly lethal malignancy. Globally, BLCA is the ninth most frequently diagnosed cancer, with approximately 614,000 new cases and 220,000 fatalities reported in 2022 1. Patients diagnosed with nonmetastatic muscle-invasive bladder cancer (MIBC) have a five-year survival rate of only 36%, which decreases drastically to 5% when the cancer has spread to distant sites 2. Chemotherapy combined with surgical resection is the standard treatment for MIBC patients. However, chemotherapy resistance frequently occurs in patients with resectable and advanced BLCA, leading to cancer recurrence, metastasis, and poor survival rates 3. Thus, overcoming treatment resistance remains a critical barrier to curing BLCA patients 4. Currently, gemcitabine (GEM) is one of the frontline therapies for BLCA chemotherapy, with only 56% of non-muscle-invasive bladder cancer (NMIBC) patients showing clinical benefit 5. Moreover, it has limited efficacy in reducing the risk of progression to MIBC. Despite its pivotal role in first-line chemotherapy for MIBC over the years, resistance to GEM in BLCA has not been thoroughly researched. These unsatisfactory results highlight the critical necessity of deciphering the mechanisms behind chemotherapy resistance in BLCA and pinpointing alternative therapeutic targets that may improve treatment efficacy.

The Warburg effect plays a pivotal role in fostering chemotherapy resistance in tumors 6. It describes how cancer cells generate energy through glycolysis even in the presence of oxygen, resulting in increased lactate production 7, 8. Understanding the molecular mechanisms underlying this phenomenon is crucial for the development of new therapeutic strategies, given its hallmark significance in cancer 9. The Warburg effect enhances tumor characteristics linked to resistance to therapeutic agents, including increased drug efflux, improved DNA damage repair mechanisms, modifications in drug metabolism, epigenetic changes, mutations affecting drug targets, activation of survival pathways, and mechanisms that evade apoptosis and immune responses 10. Disruptions in the glycolytic pathway are strongly associated with acquired resistance in BLCA 11. It has been shown that an increase in ENO1, one of the key glycolytic proteins, can lead to resistance to chemotherapy including GEM and cisplatin 12-14. Specifically, alterations in lactate production and the regulatory enzyme LDHA are pivotal factors contributing to tumor cell resistance to chemotherapy 15-18. Therefore, unraveling the mechanisms underlying glycolytic changes during GEM treatment for BLCA may provide insights into the development of chemotherapy resistance and offer strategies to overcome the carcinoma.

DLGAP5 (Discs large homolog-associated protein 5) is a microtubule-associated protein known for stabilizing K-fibers and facilitating chromosome aggregation by regulating Kif18A dynamics at kinetochore microtubules 19-21. Its high expression in endometrial cancers is correlated with poor patient prognosis. In these tumors, *DLGAP5* knockdown suppresses Wnt/β-catenin signaling, reduces proliferation, induces apoptosis, and hinders invasion 22. Additionally, DLGAP5 increases the growth and movement of gallbladder cancer cells and influences M2 macrophage polarization via the cAMP signaling pathway 23. In prostate cancer, it stabilizes the p53 and ATM proteins, thereby inhibiting the apoptosis induced by γ-rays 24. However, the understanding of the role of DLGAP5 in BLCA remains limited 25-28. Our last publication demonstrated that high DLGAP5 expression in BLCA correlates with poor clinical outcomes 29. Building on that finding, the current study further reveals that DLGAP5 potentially affects BLCA cell chemosensitivity to GEM, although the exact mechanism remains to be elucidated.

Here, we underscore the pivotal role of DLGAP5 in regulating glycolytic activity in BLCA and enhancing resistance to GEM chemotherapy. Further validation studies revealed the interaction of DLGAP5 with MYC and its facilitation of MYC deubiquitination via USP11, thereby increasing glycolytic levels in BLCA cells. This reciprocal reinforcement among DLGAP5, USP11, and MYC substantially contributes to BLCA resistance. Therefore, our research identifies DLGAP5 as a potential biomarker for predicting chemotherapy response and proposes targeting the DLGAP5/MYC/glycolysis metabolic pathway as a strategy to overcome GEM resistance in BLCA.

## Results

### DLGAP5 enhances GEM resistance in BLCA cells

**Figure 1:**
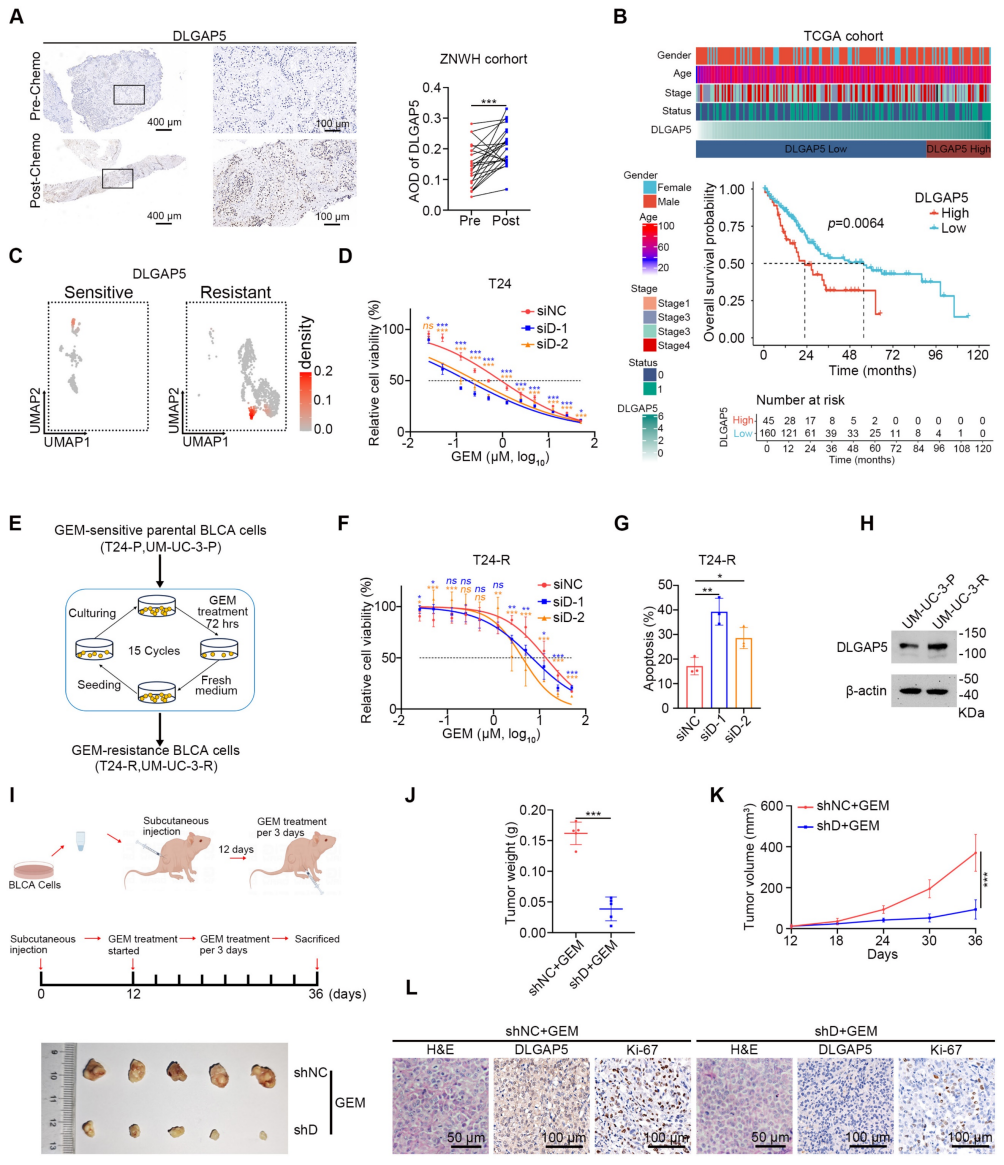
** DLGAP5 enhances GEM resistance in BLCA cells.** (**A**) Representative images (left panel) and statistical values (right panel) of IHC staining analysis of DLGAP5 protein levels in patients with BLCA (ZNWH cohort) treated with GEM chemotherapy (*n* = 24, ZNWH cohort_BCLA subgroup, details in Table S2). (**B**) Overall survival analysis of chemotherapy patients with different *DLGAP5* mRNA levels in the TCGA BLCA dataset (*n* = 412). The optimal cut-point of the DLGAP5 mRNA expression was cut-off value. Patients with missing survival data were not included. The statistical significance of the survival data was ascertained by the log-rank test of Kaplan-Meier analysis. (**C**) Uniform Manifold Approximation and Projection (UMAP) visualization showing the expression levels of DLGAP5 in the epithelial cells of chemosensitive and chemoresistant tumors from a MIBC patient (GSE192575). (**D**) Cell viability of T24 cells with *DLGAP5* knockdown after 48 h of treatment with various concentrations of GEM, as measured by MTT assay (*n* = 6). (**E**) Schematic overview showing the establishment of GEM-resistant cell lines (T24-R, UM-UC-3-R). (**F**) MTT assay results showing the viability of T24-R cells with *DLGAP5* knockdown after 48 h of exposure to different concentrations of GEM (*n* = 6). (**G**) Statistical analysis of apoptosis in T24-R cells with *DLGAP5* knockdown after 48 h of 10 μM GEM treatment (*n* = 3). (**H**) Western blot analysis of DLGAP5 proteins in UM-UC-3-P and UM-UC-3-R cells. (**I**) *In vivo* model construction and drug treatment (top). General view of dissected tumors from each group (bottom). (**J**) Weights of the tumors in each group (*n* = 5) after the tumors were surgically dissected. (**K**) Tumor growth of the indicated grafted mice treated with GEM was measured (*n* = 5). (**L**) Representative H&E (Scale bar = 100 μm) and IHC (Scale bar = 50 μm) staining analysis of subcutaneous tumor tissues from the xenograft models. Statistical significance of data was ascertained by two-tailed paired Student's t-test (A), two-tailed unpaired Student's t-test (J, K), and one-way ANOVA with Tukey's multiple comparisons test analyses (D, F, G). All statistical data are presented as mean ± SD, * *p* < 0.05, ** *p* < 0.01, *** *p* < 0.001.

Our previous publication showed that DLGAP5 is upregulated in BLCA tissues, positively correlates with the clinical stage of the disease, and significantly impacts prognosis 29. Given the potential role of DLGAP5 in the progression of BLCA, we found it necessary to further investigate its function in the chemotherapy response of BLCA cells. We collected tumor samples from 24 BLCA patients who underwent GEM-based chemotherapy, both before and after treatment. Immunohistochemical (IHC) staining revealed increased DLGAP5 expression in post-treatment tumor samples compared to pre-treatment samples, suggesting a critical role for DLGAP5 in GEM resistance of BLCA (Figure 1A). An examination of the TCGA database revealed that individuals exhibiting elevated *DLGAP5* levels experienced significantly poorer overall survival rates post-chemotherapy than those with lower *DLGAP5* expression (Figure 1B and Table S1). Single-cell RNA sequencing data (GSE192575) from MIBC patients, both treated and not treated with chemotherapy, revealed high *DLGAP5* expression in resistant epithelial cells (Figure 1C and Figure S1A-B).

Furthermore, we observed that knocking down *DLGAP5* markedly increased the sensitivity of BLCA cells to GEM, whereas overexpressing DLGAP5 markedly decreased GEM sensitivity (Figure 1D and Figure S2A-G). Additionally, *DLGAP5* silencing enhanced GEM-induced apoptosis in BLCA cells, whereas DLGAP5 overexpression reduced apoptosis (Figure S2H-K). In addition to GEM, we investigated the effect of *DLGAP5* knockdown in BLCA cells treated with cisplatin (CIS) by assessing cell viability and apoptosis. The results demonstrated that knocking down *DLGAP5* increased the sensitivity of BLCA cells to CIS (Figure S3A-D). In summary, our research indicates that lowering DLGAP5 levels increases the chemosensitivity of BLCA cells.

To further investigate the role of DLGAP5 in GEM resistance, we developed GEM-resistant T24 and UM-UC-3 cell lines (T24-R and UM-UC-3-R) via a cycle of GEM stimulation, passaging, and restimulation (Figure 1E) and the IC50 values were elevated from 0.77 μM to 12.06 μM in T24 cells and from 0.32 μM to 9.51 μM in UM-UC-3 cells (Figure S4A-B). Consistent with our previous observations, *DLGAP5* silencing increased the sensitivity of these GEM-resistant cells and increased GEM-induced apoptosis (Figure 1F-G and Figure S4C-E). We additionally noted a substantial increase in both DLGAP5 transcription and protein levels in the GEM-resistant cells (Figure 1H and Figure S4F-H). GEM treatment of BLCA cells induced a similar increase in DLGAP5 expression (Figure S4I-L). Importantly, re-expressing DLGAP5 after knockdown effectively reversed the enhanced GEM sensitivity caused by *DLGAP5* depletion. These findings indicate that DLGAP5 plays a crucial role in promoting GEM resistance in BLCA (Figure S4M-P).

Furthermore, in a mouse subcutaneous xenograft model, tumors formed from *DLGAP5*-silenced cells exhibited greater sensitivity to GEM, as evidenced by reduced tumor size, weight, growth rate, and lower Ki-67 levels compared to control groups. (Figure 1I-L and Figure S4Q). To further investigate the role of Dlgap5 in GEM resistance under more physiologically relevant conditions, we generated a *Dlgap5* knockout (*Dlgap5*^-/-^) mouse model (Figure S5A). Using BBN (N-butyl-N-(4-hydroxybutyl) nitrosamine) induction, we established a spontaneous BLCA model 30, 31 (Figure S5B). Results showed that, compared to wild-type (WT) mice and those treated with GEM alone, the *Dlgap5*^-/-^ mice treated with GEM exhibited reduced tumorigenesis and malignancy (Figure S5C). Importantly, no significant differences in body weight were observed between *Dlgap5*^-/-^ and WT mice during the study. Additionally, H&E staining of major organs (heart, liver, spleen, lung, and kidney) indicated no apparent adverse effects from *Dlgap5* deletion (Figure S5D-F).

These results demonstrate that DLGAP5 expression is closely associated with GEM sensitivity in BLCA, with higher DLGAP5 levels contributing to chemotherapy resistance in both mouse model and patients. This study lays the groundwork for potential DLGAP5-targeted therapies.

### DLGAP5 influences GEM resistance in BLCA by regulating glycolysis

**Figure 2:**
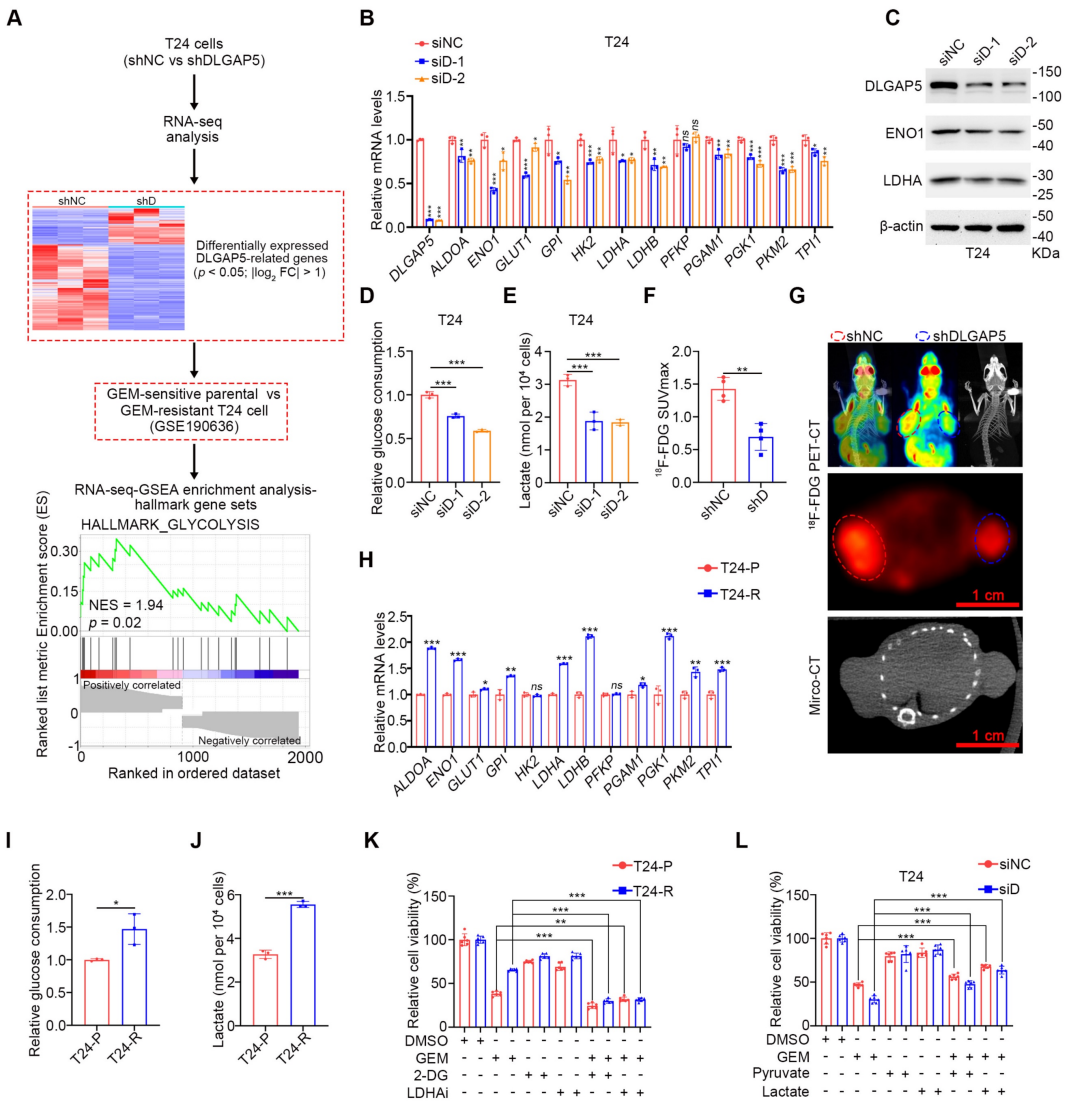
** DLGAP5 influences GEM resistance in BLCA by regulating glycolysis.** (**A**) Schematic of the identification of GEM resistance-related genes. Following dataset intersection, GSEA enrichment analysis revealed significant enrichment of the glycolysis signaling pathway. (**B**) Changes in the transcription levels of glycolysis-related genes in T24 cells after *DLGAP5* knockdown (*n* = 3). (**C**) Western blot analysis of the effects of *DLGAP5* knockdown on ENO1 and LDHA protein expression in T24 cells. Relative glucose uptake (**D**) and intracellular lactate production (**E**) in T24 cells after *DLGAP5* knockdown (*n* = 3). Subcutaneous tumor models of BLCA were established in BALB/c nude mice via the injection of T24 control cells or T24 cells with *DLGAP5* knockdown. Glucose uptake was analyzed via ^18^F-FDG PET-CT imaging (*n* = 4). Statistical values of SUVmax (**F**) and representative PET-CT imaging (**G**). Scale bar = 1 cm. (**H**) mRNA levels of glycolysis-related genes in T24-P and T24-R cells (*n* = 3). Relative glucose uptake (**I**) and intracellular lactate production (**J**) in T24-P and T24-R cells (*n* = 3). (**K**) T24-P and T24-R cells were treated with the indicated combinations of GEM (10 μM), 2-DG (2 mM), and oxamate (10 mM) before cell viability was measured at 48 h (*n* = 6). (**L**) siNC and siDLGAP5 T24 cells were treated with the indicated combinations of GEM (1 μM), pyruvate (2 mM), and lactate (10 mM) before cell viability was measured at 48 h (*n* = 6). Statistical significance of data was ascertained by two-tailed unpaired Student's t-test (F, H-L) and one-way ANOVA with Tukey's multiple comparisons test analyses (B, D, E). All statistical data are presented as mean ± SD, * *p* < 0.05, ** *p* < 0.01, *** *p* < 0.001.

To further investigate how DLGAP5 affects GEM resistance in BLCA, we knocked down *DLGAP5* in T24 cells and performed RNA-seq analysis (GSE241523). We identified 2464 differentially expressed DLGAP5-related genes. The genes were intersected with RNA-seq data from GEM-resistant T24 cells (GSE190636) and subjected to Gene Set Enrichment Analysis (GSEA). Analysis revealed notable enrichment of the glycolysis signaling pathway among these DLGAP5-related resistance genes (Figure 2A). Knocking down *DLGAP5* in BLCA cells resulted in a noticeable reduction in the expression of most glycolysis-related genes (Figure 2B and Figure S6A). Western blot analysis confirmed that the decrease in DLGAP5 led to reduced protein levels of two key glycolytic enzymes, ENO1 and LDHA (Figure 2C and Figure S6B).

To verify that these changes were sufficient to alter glycolytic flux, we examined metabolite levels in the glycolysis pathway following *DLGAP5* knockdown. The results revealed reduced glucose consumption, decreased intracellular levels of lactate and pyruvate, and significantly decreased lactate dehydrogenase (LDH) activity in BLCA cells (Figure 2D-E and Figure S6C-H). In a mouse xenograft model, *DLGAP5* knockdown significantly reduced ^18^F fluoro-D-deoxyglucose (^18^F-FDG) uptake which accurately reflects the level of glucose metabolism in tumor tissues and inhibited tumor growth (Figure 2F-G).

Further analysis of glycolysis-related gene expression in the GEM-resistant cell lines revealed that most glycolysis-related genes were upregulated in the GEM-resistant cells (Figure 2H and Figure S6I). Additionally, the protein levels of the key glycolytic enzymes ENO1 and LDHA were greater in GEM-resistant cells than in parental cells (Figure S6J). These cells also exhibited increased glucose uptake, higher intracellular levels of lactate and pyruvate, and elevated LDH activity than GEM-sensitive parental cells did (Figure 2I-J and Figure S6K-P).

Next, we explored the relationship between glycolysis and GEM resistance in BLCA. The sensitivity of BLCA cells to GEM was significantly greater in low-glucose medium (1500 mg/L) than in high-glucose medium (4500 mg/L) (Figure S6Q), suggesting a critical role for glucose metabolism levels in GEM resistance. Lactate, produced as a result of the Warburg effect, is known to enhance tumor cell resistance in various cancers 16, 17, 32, 33. To determine whether GEM resistance in BLCA is related to lactate production, we treated GEM-resistant BLCA cells with the glycolysis inhibitor 2-DG or the LDHA inhibitor oxamic acid sodium, both of which are well-recognized inhibitors that have been used in numerous studies 15, 34-41. Both treatments increased GEM sensitivity in GEM-sensitive parental and GEM-resistant cells and reduced the difference in sensitivity between the two (Figure 2K and Figure S6R). Moreover, the inhibition of intracellular glucose metabolism by 2-DG can effectively reduce the GEM resistance of BLCA cells caused by DLGAP5 overexpression (Figure S6S-T). Importantly, the addition of exogenous glycolytic products, particularly lactate, effectively reversed the increased GEM sensitivity caused by *DLGAP5* knockdown in BLCA cells (Figure 2L and Figure S6U-W). In summary, DLGAP5 influences GEM resistance in BLCA cells by regulating the glycolysis pathway.

### The role of MYC in DLGAP5-mediated GEM resistance

**Figure 3:**
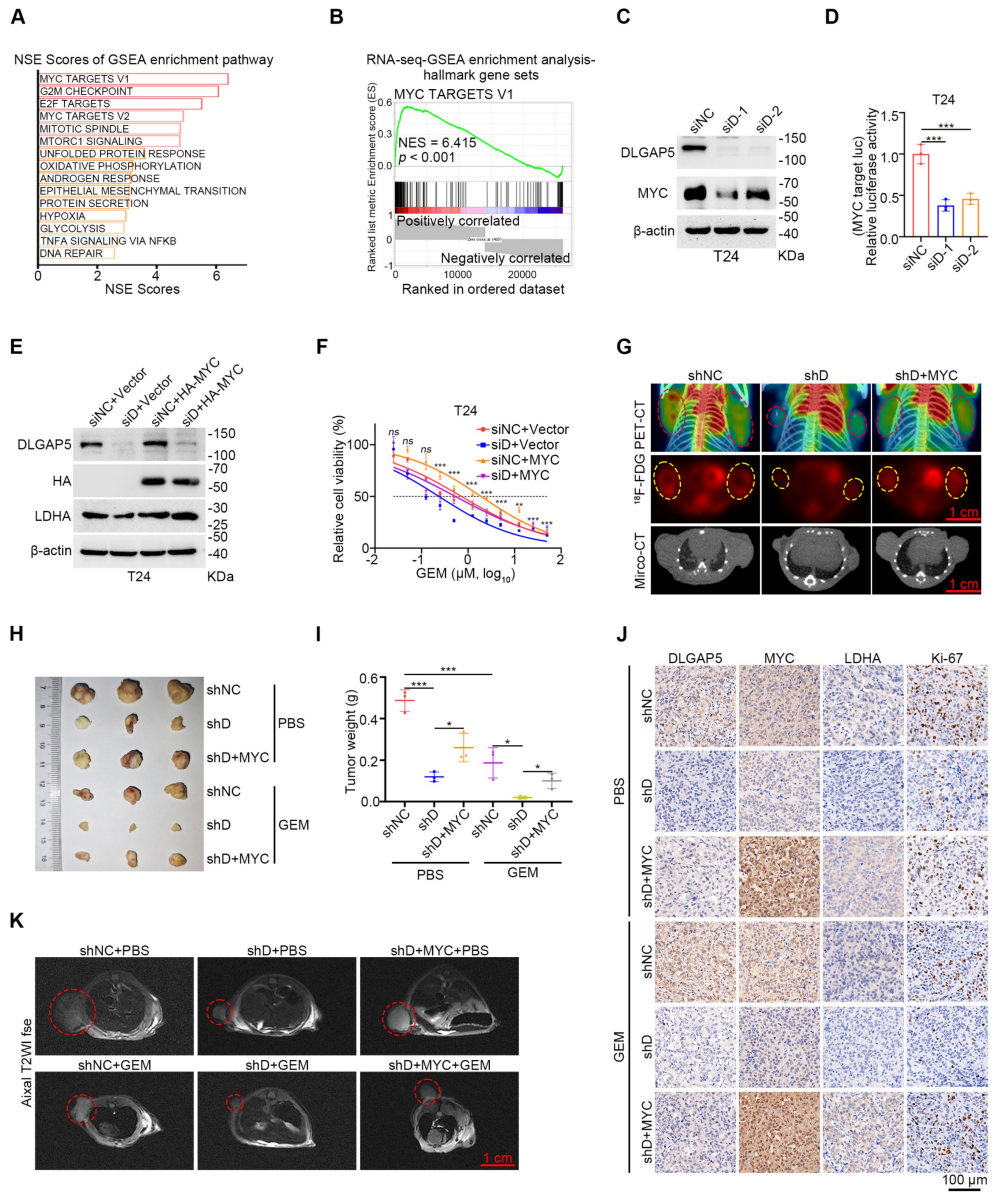
** The role of MYC in DLGAP5-mediated GEM resistance.** (**A**) Hallmark gene sets (*https://www.gsea-msigdb.org/gsea/msigdb*) related to DLGAP5 from enrichment analysis of the gene expression matrix from RNA-seq assays. GSEA was performed with the R package “clusterProfiler”. *p*-value was computed via two-tailed Fisher's exact test. The Benjamini-Hochberg method was used to adjust the *p*-value. The top 15 gene sets were selected on the basis of the lowest *p*.adjust values and sorted in ascending order on the basis of the normalized enrichment score (NES), from largest to smallest. (**B**) GSEA of *DLGAP5* knockdown in the hallmark MYC TARGET V1 gene set. (**C**) Western blot analysis of the effects of *DLGAP5* knockdown on MYC proteins in T24 cells. (**D**) T24 cells were transfected with siDLGAP5 for 24 h, transfected with 5× E-box luciferase reporter for 48 h, and finally subjected to a dual-luciferase reporter assay (*n* = 3). (**E**) Western blots showing the protein expression of DLGAP5, HA-MYC, and LDHA in T24 cells after *DLGAP5* knockdown and MYC overexpression. (**F**) Viability of T24 cells treated with various concentrations of GEM for 48 h, as determined via the MTT assay (*n* = 6). The asterisk indicates statistical significance between siD+Vector and siD+MYC. (**G**) Subcutaneous tumor models of BLCA were established in BALB/c nude mice via the injection of T24 shNC cells, T24 shDLGAP5 cells and T24 shDLGAP5+MYC cells. Glucose uptake was analyzed via ^18^F-FDG PET-CT imaging (*n* = 6). Scale bar = 1 cm. (**H**) General view of dissected tumors of each group (*n* = 3). (**I**) Weights of the tumors in each group after the tumors were surgically dissected (*n* = 3). (**J**) Representative IHC staining analysis of subcutaneous tumor tissues from xenograft models. Scale bar = 100 μm. (**K**) Representative MRI images of axial T2, showing the subcutaneous tumor xenografts. Scale bar = 1 cm. Statistical significance of data was ascertained by one-way ANOVA with Tukey's multiple comparisons test analyses (D, F, I). All statistical data are presented as mean ± SD, * *p* < 0.05, ** *p* < 0.01, *** *p* < 0.001.

Further analysis of RNA-seq data from shDLGAP5-treated cells revealed a significant enrichment of MYC target signaling pathways (including MYC target V2 and V1) following *DLGAP5* knockdown, suggesting that DLGAP5 may regulate MYC-related downstream signals (Figure 3A-B and Figure S7A-C). Additionally, we found that *DLGAP5* knockdown significantly reduced MYC protein levels, while overexpression of DLGAP5 increased MYC abundance in BLCA cells (Figure 3C and Figure S7D-E). As a crucial transcription factor in tumor metabolism, MYC controls the transcription of most glycolysis genes, thereby influencing the glycolytic process in tumor cells 42, 43. We subsequently utilized a dual-luciferase reporter plasmid containing a 5× E-box sequence to assess MYC transcriptional activity 44. Following *DLGAP5* knockdown, MYC transcriptional activity was markedly reduced (Figure 3D and Figure S7F). Additionally, reducing MYC levels effectively enhanced GEM sensitivity in both GEM-sensitive parental and GEM-resistant BLCA cells (Figure S8A-F). To further confirm the role of MYC, we overexpressed MYC in *DLGAP5*-knockdown cells and assessed their GEM sensitivity. As expected, MYC overexpression rescued the effects of *DLGAP5* knockdown, including increased LDHA expression, reduced GEM-induced apoptosis, and decreased GEM sensitivity (Figure 3E-F and Figure S8G-J). Meanwhile, we knocked down *MYC* in DLGAP5-overexpress cells. Knocking down *MYC* counteracted the GEM resistance caused by DLGAP5 (Figure S8K-N). Additionally, positron emission tomography (PET)-CT images revealed that MYC overexpression counteracted the reduced uptake of ^18^F-FDG caused by *DLGAP5* knockdown (Figure 3G and Figure S8O). These findings indicate that DLGAP5 enhances downstream glucose metabolism and GEM resistance through the MYC pathway.

*In vivo* validation using a mouse subcutaneous xenograft model revealed that *DLGAP5* knockdown in T24 tumors enhanced GEM sensitivity and reduced the expression of the cell proliferation marker Ki-67. The overexpression of MYC counteracted the increased chemotherapy sensitivity caused by *DLGAP5* knockdown (Figure 3H-K and Figure S8P-R). In conclusion, these data demonstrate that MYC is critical for the DLGAP5-mediated promotion of GEM resistance.

### DLGAP5 deubiquitinates and stabilizes MYC

**Figure 4:**
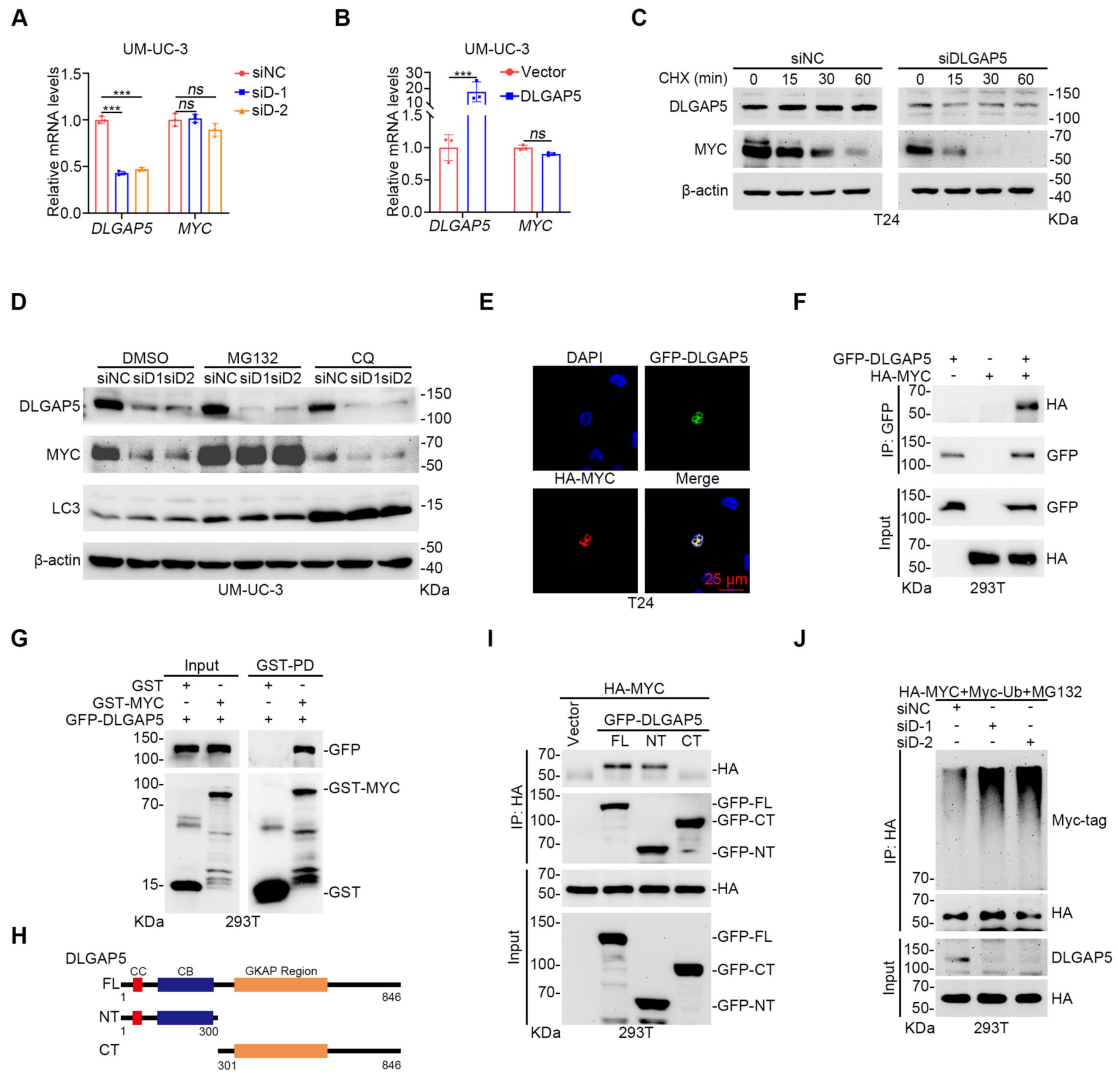
** DLGAP5 deubiquitinates and stabilizes MYC.** After DLGAP5 was knocked down (**A**) or overexpressed (**B**) in UM-UC-3 cells, the mRNA level was detected via qRT-PCR (*n* = 3). (**C**) Western blot analysis of the effect of *DLGAP5* knockdown on MYC degradation in T24 cells incubated with CHX (50 μg/mL) for the indicated time points. (**D**) UM-UC-3 cells were transfected with siNC or siDLGAP5 for 48 h and then treated with DMSO or MG132 (10 μM) or CQ (100 μM) for 8 h before lysis. Protein levels were analyzed by Western blotting. (**E**) Confocal imaging confirming that DLGAP5 co-localized with MYC in the nucleus of T24 cells. (**F**) Co-IP assay showing that exogenous DLGAP5 interacts with MYC in 293T cells. (**G**) 293T cells were transfected with GFP-DLGAP5 for 48 h, and a GST pull-down assay revealed that DLGAP5 interacts with MYC *in vitro*. Scale bar = 25 μm. (**H**) Schematic representation of various DLGAP5 truncations. (**I**) Co-IP assay showing that DLGAP5-NT interacts with MYC in 293T cells. (**J**) 293T cells were transfected with the specified plasmids for 48 h, followed by an 8 h of treatment with 10 μM MG132. Western blots showing exogenous ubiquitination of MYC after *DLGAP5* knockdown in 293T cells. Statistical significance of data was ascertained by two-tailed unpaired Student's t-test (B) and one-way ANOVA with Tukey's multiple comparisons test analyses (A). All statistical data are presented as mean ± SD, * *p* < 0.05, ** *p* < 0.01, *** *p* < 0.001.

To investigate how DLGAP5 regulates MYC expression, we first examined its impact on MYC transcription levels. Quantitative reverse transcription-PCR (qRT-PCR) experiments revealed that altering DLGAP5 expression, either by overexpression or knockdown, did not affect *MYC* mRNA levels (Figure 4A-B and Figure S9A-B), indicating that DLGAP5 likely regulates MYC through posttranslational modifications. Using cycloheximide (CHX) to inhibit intracellular protein synthesis, we found that *DLGAP5* knockdown significantly accelerated MYC protein degradation, whereas the overexpression of DLGAP5 increased MYC stability (Figure 4C and Figure S9C-E). Additionally, increasing DLGAP5 expression led to a dose-dependent increase in MYC levels (Figure S9F). Given that MYC protein stability is regulated primarily via the ubiquitin-proteasome pathway, we further investigated this mechanism 45-48. The addition of the proteasome inhibitor MG132 (not the autophagy inhibitor chloroquine) almost completely rescued the reduction in MYC protein levels caused by *DLGAP5* deficiency (Figure 4D and Figure S9G), suggesting that DLGAP5 stabilizes MYC by modulating the ubiquitin-proteasome pathway.

Immunofluorescence co-localization experiments revealed that DLGAP5 and MYC co-localize in the nucleus (Figure 4E and Figure S9H). Co-immunoprecipitation (co-IP) experiments verified the interaction between DLGAP5 and MYC (Figure 4F and Figure S9I). GST pull-down experiments additionally confirmed that MYC directly interacts with DLGAP5 (Figure 4G). To identify the precise binding regions between MYC and DLGAP5, we constructed truncation mutants and discovered that MYC attaches to the N-terminal of DLGAP5 (amino acids 1-300), whereas DLGAP5 associates with both the N-terminal and C-terminal of MYC (Figure 4H-I and Figure S9J-K). Ubiquitination assays revealed that *DLGAP5* knockdown increased the level of MYC protein ubiquitination, whereas DLGAP5 overexpression decreased it (Figure 4J and Figure S9L). Further investigations using co-transfected ubiquitin mutants with MYC revealed that *DLGAP5* knockdown specifically increased the K11-linked polyubiquitination of MYC, indicating that DLGAP5 stabilizes MYC through K11-linked polyubiquitin chains (Figure S9M). These findings indicate that DLGAP5 stabilizes the MYC protein through the ubiquitin-proteasome pathway.

### The deubiquitinating enzyme USP11 improves MYC stability and promotes GEM resistance

**Figure 5:**
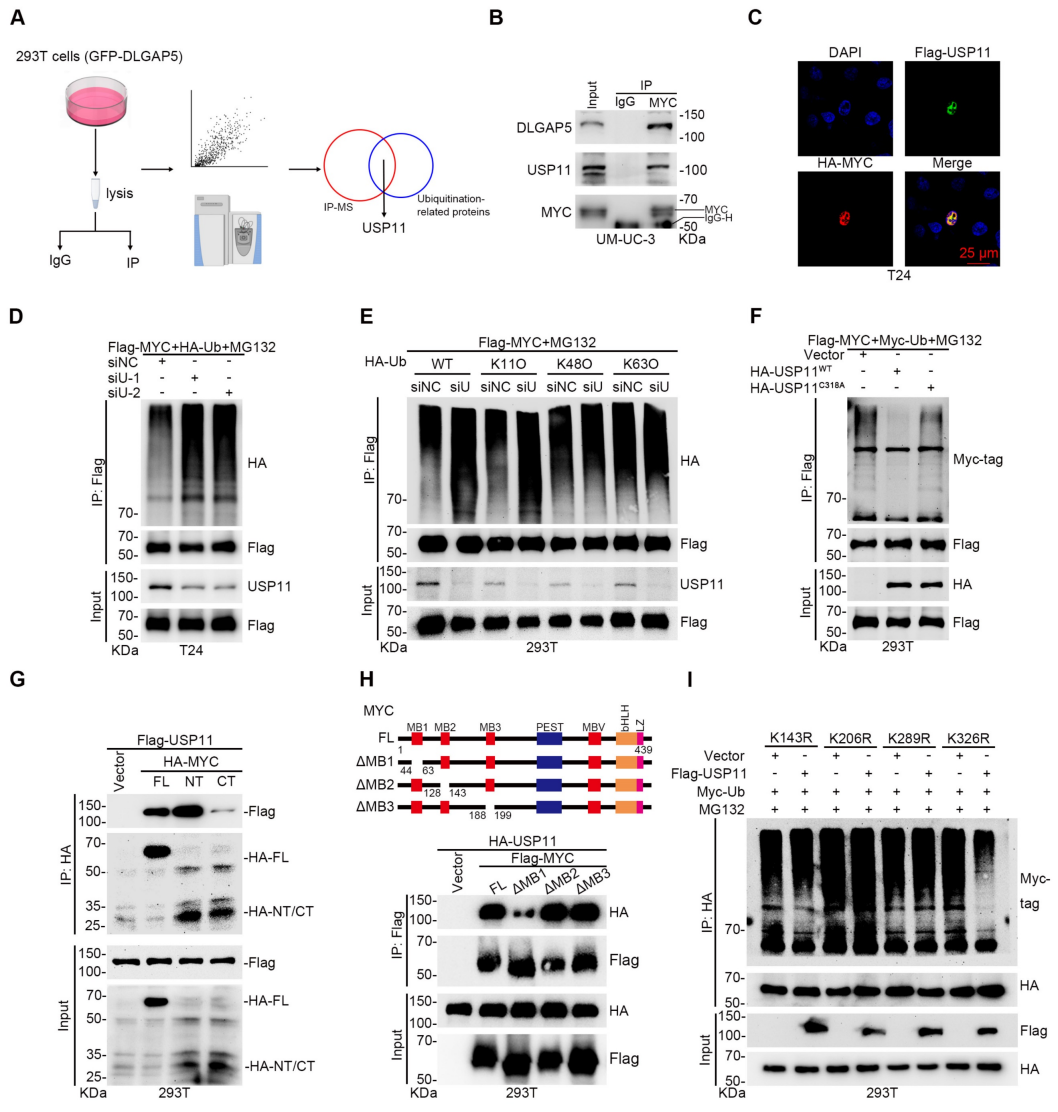
** The deubiquitinating enzyme USP11 regulates MYC stability and promotes GEM resistance.** (**A**) Flow diagram showing the IP of DLGAP5 and subsequent LC-MS/MS analysis. (**B**) Co-IP assay showing that endogenous DLGAP5 and USP11 interact with MYC in UM-UC-3 cells. (**C**) Confocal imaging confirming that USP11 co-localized with MYC in the nucleus of T24 cells. The scale bar is 25 μm. (**D**) T24 cells were transfected with the specified plasmids for 48 h, followed by an 8 h of treatment with 10 μM MG132. Western blots showing exogenous ubiquitination of MYC after *USP11* knockdown in 293T cells. (**E**) 293T cells were transfected with the specified plasmids for 48 h, followed by an 8 h of treatment with 10 μM MG132. Ubiquitination assays were conducted to examine the specific ubiquitin chain linkages catalyzed by USP11 on MYC proteins. (**F**) 293T cells were transfected with the specified plasmids for 48 h, followed by an 8 h of treatment with 10 μM MG132. Western blots showing exogenous ubiquitination of MYC after USP11 (WT) and USP11 (C318A) were overexpressed in 293T cells. (**G**) Co-IP assays showing that USP11 binds to MYC, particularly the N-terminal domain in 293T cells. (**H**) Schematic representation of various MYC deletion mutations (top) and co-IP assays showing that USP11 interacts with the MYC-NT MB1 domain in 293T cells (bottom). (**I**) 293T cells were transfected with the specified plasmids for 48 h, followed by an 8 h of treatment with 10 μM MG132. Ubiquitination experiments were conducted to identify the specific lysine residues on MYC that are deubiquitinated by USP11.

Given that DLGAP5 is a microtubule-associated protein lacking known enzymatic activity in ubiquitination regulation, we hypothesized that it might influence MYC ubiquitination by affecting ubiquitination-associated enzymes. To evaluate this, we overexpressed DLGAP5 in 293T cells and identified several ubiquitination-associated enzymes that interact with DLGAP5 29 (Figure 5A). Among these, USP11 scored the highest and is known to interact with MYC, although further studies are limited 49.

Focusing on the interaction between USP11 and MYC, we conducted co-IP experiments and confirmed that USP11 interacts with MYC (Figure S10A-B). Endogenous co-IP revealed that MYC binds with both DLGAP5 and USP11 in BLCA cells (Figure 5B and Figure S10C). GST pull-down experiments additionally confirmed a direct binding between the USP11 and MYC proteins (Figure S10D). Immunofluorescence experiments revealed the co-localization of MYC and USP11 in the nucleus (Figure 5C and Figure S10E). By altering USP11 expression, we found that USP11 regulates MYC protein levels (Figure S10F-H). CHX and ubiquitination assays revealed that USP11 stabilizes MYC via deubiquitination, specifically through K11-linked polyubiquitin chains (Figure 5D-E and Figure S10I-O).

To confirm the enzymatic role of USP11, we created a USP11 C318A catalytically inactive mutant plasmid. Compared with wild-type USP11, this mutant failed to regulate MYC protein stability and ubiquitination (Figure 5F and Figure S10P-S). Co-IP assays using various MYC truncation mutants revealed that MYC interacts with the M3 region (amino acids 504-963) of USP11, whereas USP11 binds to both the N-terminal (amino acids 1-221) and C-terminal (amino acids 220-439) regions of MYC (Figure 5G and Figure S11A), particularly the N-terminal domain. Earlier studies revealed that the N-terminal region of the MYC protein facilitates its binding with various proteins and controls its stability 29, 44, 50. Here, we discovered that the MYC mutant lacking the MB1 domain (N-terminal domain) exhibited significantly weakened interaction with USP11 and that USP11 could not regulate the stability or polyubiquitination of this mutant (Figure 5H and Figure S11B-D). To pinpoint the lysine residues on MYC regulated by USP11, we mutated several lysines to arginines and performed ubiquitination assays. We found that USP11 does not affect the polyubiquitination or protein levels of the MYC mutants K143R, K206R, and K289R (Figure 5I and Figure S11E-J). These results suggest that USP11 stabilizes MYC by interacting with its N-terminal MB1 domain and regulating K11-linked polyubiquitination at K143, K206, and K289.

Given that USP11 effectively stabilizes MYC in BLCA cells, we explored its role in GEM resistance. Knockdown of *USP11* significantly increased GEM sensitivity and apoptosis in BLCA cells, whereas USP11 overexpression had the opposite effect (Figure S12). Additionally, *USP11* knockdown reduced glycolysis in BLCA cells, which was reversed by the addition of lactate and pyruvate (Figure S13A-J). The overexpression of MYC effectively reversed the increased GEM sensitivity and induced apoptosis caused by *USP11* knockdown (Figure S13K-O). These findings indicate that USP11 promotes GEM resistance in BLCA cells by increasing MYC stability.

### The DLGAP5-USP11-MYC feedback loop induces GEM resistance in BLCA cells

**Figure 6:**
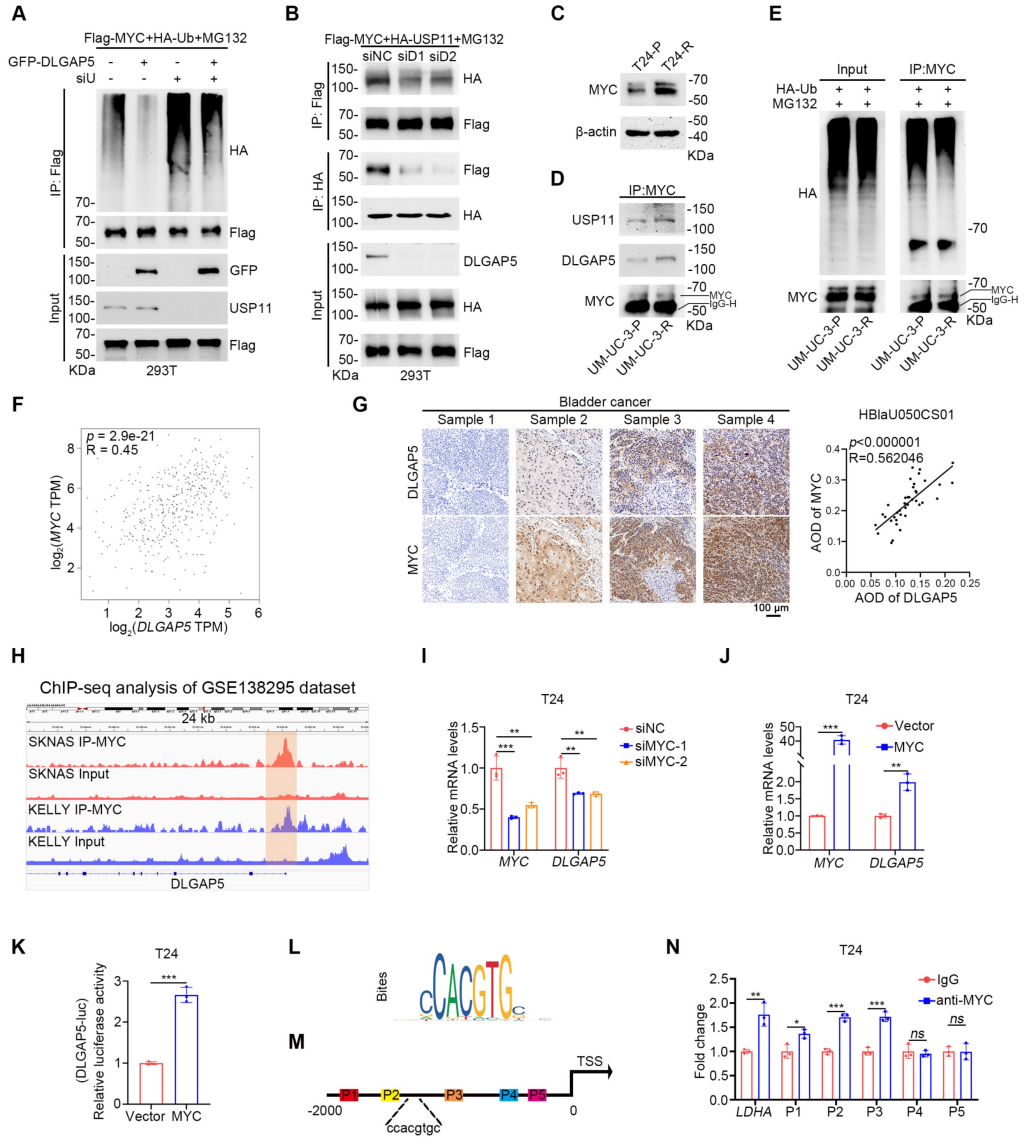
** The DLGAP5-USP11-MYC feedback loop induces GEM resistance in BLCA cells.** (**A**) 293T cells were transfected with the specified plasmids for 48 h, followed by treatment with 10 μM MG132 for 8 h. Western blots showed changes in exogenous ubiquitination of MYC. (**B**) Co-IP assays demonstrated that exogenous USP11 and MYC interactions decreased with *DLGAP5* knockdown. (**C**) Western blot analysis of MYC proteins in T24-P and T24-R cells. (**D**) Co-IP assay showing that endogenous DLGAP5 and USP11 interact with MYC in UM-UC-3-P and UM-UC-3-R cells. (**E**) UM-UC-3-P and UM-UC-3-R cells were transfected with the HA-Ub for 48 h, followed by an 8 h of treatment with 10 μM MG132. Ubiquitination experiments evaluating the ubiquitination levels of MYC. (**F**) Spearman correlation analysis of the expression levels of *DLGAP5* and *MYC* in the GEPIA database. (**G**) Representative images of IHC staining of DLGAP5 and MYC in human BLCA specimens from a BLCA tissue microarray (HBlaU050CS01). Scale bars, 100 μm. Pearson correlation analysis was used to determine the degree of association between DLGAP5 and MYC via IHC staining (*n* = 40). *p*-value was obtained by Student's t-test. (**H**) Genome browser tracks of MYC occupancy at the DLGAP5 locus in SKNAS and KELLY cells (GSE138295). The genome browser map is displayed via IVG software. The green region marks a region in the *DLGAP5* promoter region where MYC is significantly enriched relative to the input. T24 cells with *MYC* knocked down (**I**) or MYC overexpressed (**J**), and the mRNA levels were detected by qRT-PCR (*n* = 3). (**K**) Dual-luciferase reporter assay of *DLGAP5* promoter activity after overexpressing MYC in T24 cells (*n* = 3). (**L**) Binding site of MYC on promoter sequences was obtained from the JASPAR database. (**M**) Schematic diagram of primers designed for ChIP-qPCR of the *DLGAP5* promoter sequence. (**N**) ChIP-qPCR analysis showed the enrichment degree of MYC in different regions of the *DLGAP5* promoter. IgG indicates the negative control (*n* = 3). Statistical significance of data was ascertained by two-tailed unpaired Student's t-test (J, K, N) and one-way ANOVA with Tukey's multiple comparisons test analyses (I). All statistical data are presented as mean ± SD, * *p* < 0.05, ** *p* < 0.01, *** *p* < 0.001.

While we identified the deubiquitinase USP11, which helps DLGAP5 improves MYC stability, the precise relationships among DLGAP5, USP11, and MYC remain unclear. Upon *USP11* knockdown, DLGAP5 could no longer increase MYC expression, and its ability to reduce MYC ubiquitination was abolished (Figure 6A and Figure S14A-B). Moreover, knockdown of *USP11* prevented DLGAP5 from enhancing GEM resistance in BLCA cells (Figure S14C-D), indicating that the regulation of MYC and GEM resistance by DLGAP5 is dependent on USP11. Conversely, the interaction between USP11 and MYC significantly decreased with *DLGAP5* knockdown, whereas DLGAP5 overexpression enhanced this interaction (Figure 6B and Figure S14E).

In GEM-resistant BLCA cell lines, MYC expression was also increased, similar to that of DLGAP5 (Figure 6C and Figure S15A-C). Co-IP experiments indicated that the interaction among MYC, USP11, and DLGAP5 was strengthened in GEM-resistant cells (Figure 6D). CHX assays revealed that MYC stability was greater in GEM-resistant cells than in parental cells, accompanied by a decrease in MYC ubiquitination levels (Figure 6E and Figure S15D-E).

Additionally, both GEM-stimulated and GEM-resistant cells presented increased transcription and protein levels of DLGAP5 and MYC (Figure 1H, Figure 6C, and Figure S4F-L, S15A-C, and S15F-G). The expression levels of the MYC downstream proteins LDHA, LDHB and ENO1 also increased under GEM stimulation (Figure S15F and S15H-J). Notably, the changes in MYC preceded those in DLGAP5 upon GEM stimulation (Figure S15K-N). An evaluation of the TCGA database revealed a significant correlation between *MYC* and *DLGAP5* mRNA expression in BLCA (Figure 6F). IHC analysis of 40 BLCA tissue samples from a BLCA tissue microarray (HBlaU050CS01) confirmed a positive correlation between DLGAP5 and MYC expression (Figure 6G).

Moreover, we collected BLCA patient samples, including 53 patients: 24 with paired pre- and post-GEM chemotherapy samples, 17 with only pre-chemotherapy samples, and 12 with only post-chemotherapy samples. Detailed data are shown in Table S2. A positive correlation between DLGAP5 and MYC was also confirmed in both pre- and post-chemotherapy samples (Figure S16A-C). Therefore, we hypothesize that MYC, a well-known transcription factor, may increase the transcription of DLGAP5. Public chromatin immunoprecipitation (ChIP) -seq dataset (GSE138295) revealed significant MYC binding signals in the DLGAP5 promoter region (Figure 6H). RNA-seq data (GSE225375) revealed that *MYC* knockdown reduced *DLGAP5* mRNA levels (Figure S16D). Further knockdown and overexpression experiments confirmed that MYC regulates DLGAP5 mRNA and protein levels (Figure 6I-J and Figure S16E-J). Dual-luciferase reporter assays validated the role of MYC in regulating DLGAP5 transcription (Figure 6K and Figure S16K). JASPAR database analysis supported the existence of MYC binding sites in the DLGAP5 promoter region (Figure 6L). ChIP-qPCR analysis of T24 cells confirmed MYC enrichment at the DLGAP5 promoter (Figure 6M-N).

**Figure 7:**
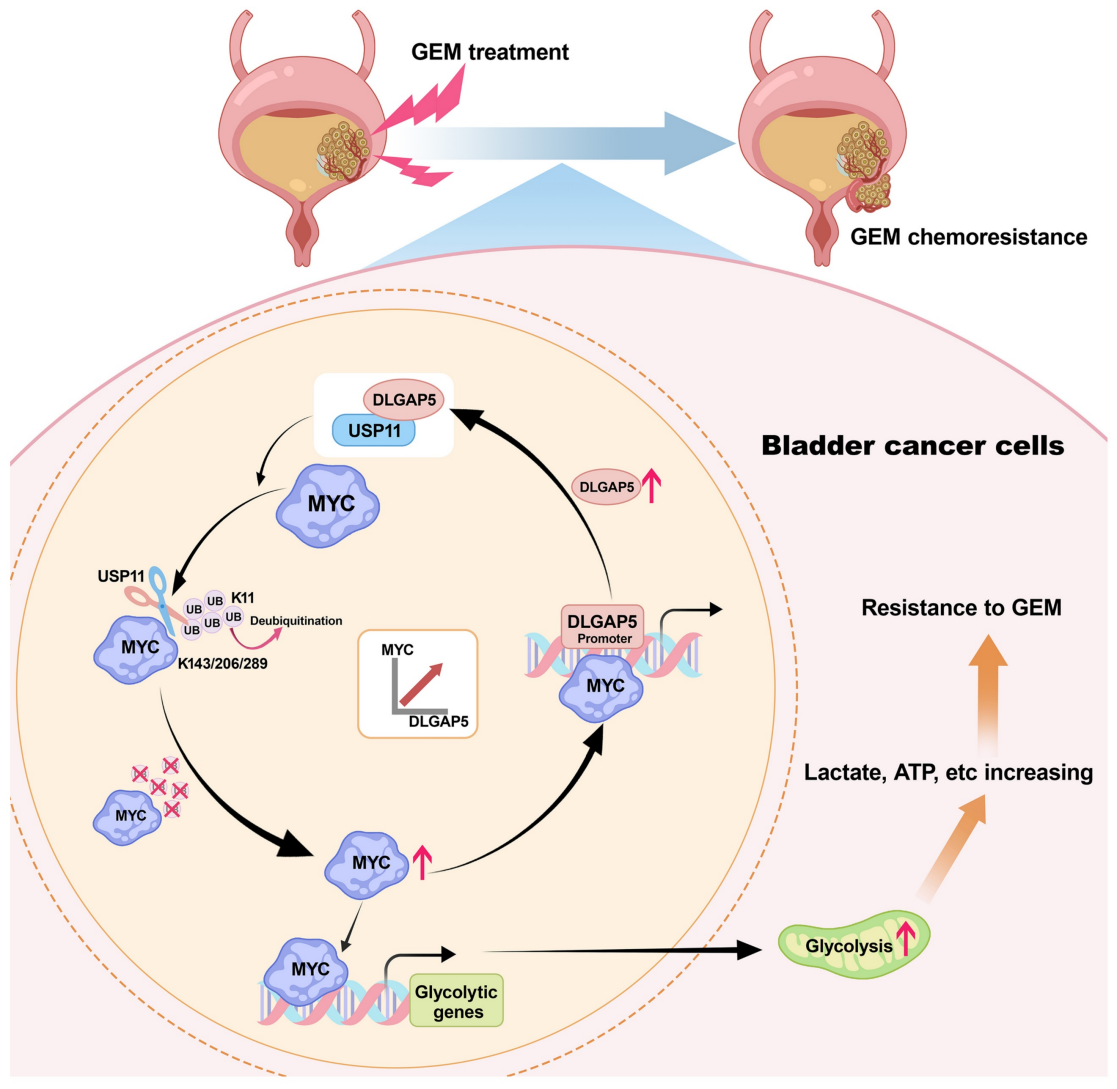
** Mechanism diagram of the study.** DLGAP5 drives BLCA GEM chemoresistance by facilitating glycolysis. In BLCA cells with high DLGAP5 levels, DLGAP5 facilitates MYC protein stability via the deubiquitinating enzyme USP11, which contributes to glycolysis. Enhanced glycolysis promotes an increase in metabolites such as lactate, leading to GEM chemoresistance. Additionally, MYC enhances DLGAP5 transcription by binding to its promoter region, forming a DLGAP5/USP11-MYC feedback loop that promotes GEM chemoresistance.

Taken together, we elucidated the interplay between DLGAP5, USP11, and MYC. MYC upregulates DLGAP5 transcriptionally, while DLGAP5 stabilizes MYC via USP11, forming a positive feedback loop that promotes GEM resistance in BLCA cells (Figure 7).

## Discussion

BLCA, the ninth most common cancer, continues to have increasing incidence and mortality rates annually 1, 51. Chemotherapy remains a critical component of first-line treatment for BLCA, but most patients experience suboptimal outcomes posttreatment, characterized by chemoresistance and tumor recurrence 38. GEM is a fundamental first-line chemotherapy drug used in various stages of BLCA treatment, including monotherapy for NMIBC, single-dose chemotherapy within 24 h perioperatively, and combination chemotherapy regimens for MIBC 2, 52, 53. However, the response rate of BLCA patients to GEM monotherapy is only 23-28% 54, 55. Therefore, understanding the mechanisms underlying GEM chemoresistance in BLCA and identifying potential molecular targets to predict and combat this resistance are crucial. In this study, we demonstrated that DLGAP5 promotes GEM chemoresistance in BLCA cells by enhancing glycolysis through a MYC-dependent mechanism.

Our previous study revealed that DLGAP5 is markedly expressed in BLCA and promotes tumor growth 29. Further clinical and experimental studies confirmed that patients with high DLGAP5 levels had poorer responses to chemotherapy and poorer survival prognosis. These findings suggest that DLGAP5 may be linked to chemoresistance in BLCA. In support of these findings, we found that knocking down *DLGAP5* markedly enhanced the sensitivity of BLCA cells to GEM, leading to increased GEM-induced apoptosis. These results expand our insight into the role of DLGAP5 in BLCA.

Recent studies have indicated that, compared with CIS, GEM-induced chemoresistance in BLCA cells is associated with significant metabolic abnormalities, particularly in glycolysis 56. By analyzing RNA-seq data from *DLGAP5*-knockdown cells and comparing them with existing RNA-seq data from GEM-resistant cells, we identified glycolysis as a key pathway upregulated by DLGAP5 in BLCA chemoresistance. The Warburg effect in tumor cells, which balances intracellular ATP and the extracellular environment, is known to support resistance to apoptosis and immune destruction, leading to chemoresistance 6. Alterations in glycolysis play crucial roles in this process. For example, the crosstalk between MUC1 and HIF-1α signaling increases glycolytic flux, leading to increased pyrimidine synthesis in tumor cells, which competes with GEM, reducing its toxicity and causing chemoresistance in pancreatic cancer 57. Another study revealed that during chemoresistance, the Warburg effect enhances DNA repair in tumor cells, enabling rapid recovery from chemo-radiotherapy damage and reducing treatment efficacy. Targeting lactate to inhibit this effect can significantly increase chemosensitivity 33. We found that treating BLCA cells with lactate counteracted the increased GEM sensitivity resulting from *DLGAP5* knockdown, supporting our conclusion.

The role of glycolysis in tumor resistance and the central role of MYC in glycolysis and cancer have drawn attention to targeting MYC and tumor metabolism 58-60. Previous studies have shown that reducing MYC levels in pancreatic cancer decreases GEM-induced neuroendocrine marker expression, increasing chemosensitivity 61. GEM treatment induces MUC5AC overexpression in pancreatic cancer, disrupting E-cadherin/β-catenin junctions and promoting β-catenin nuclear translocation, which increases MYC expression 62. The simultaneous increase in MYC and PD-L1 in GEM-resistant pancreatic cancer cells suggests that MYC may influence GEM resistance through regulating glycolysis 25. However, the roles of MYC and glycolysis in GEM resistance in BLCA are poorly understood. Additionally, the high glucose levels *in vivo* and the cellular localization of MYC and the lack of kinase-like specific activity sites make the development of drugs that target glycolysis and MYC activity challenging 42, 63, 64. Thus, methods to inhibit MYC transcription or post-translational modifications have gained increasing attention.

In this study, we discovered that DLGAP5 improves MYC protein stability through USP11, a newly identified MYC deubiquitinase. A recent article reported that USP11 can deubiquitinate and stabilize the MYC and AR proteins, thereby promoting prostate cancer progression 65. This finding further supports our findings in BLCA. Our study revealed that USP11 binds to both the N-terminal and C-terminal regions of the MYC protein, with a stronger interaction at the N-terminal domain. The conserved region within the N-terminal domain of MYC, known as the MYC box, includes several domains crucial for its function (MB1, 2, 3, and 4). Notably, MB1 is located in the transactivation domain and is essential for the transcriptional and cellular transformation activities of MYC 66. Current studies have demonstrated that various ubiquitination-related proteins, including FBW7, SKP2, FBX29, TRUSS, and HECTH9, regulate the ubiquitination of MYC by binding to the MYC box at the N-terminus, thereby controlling MYC protein abundance within cells 45, 67-70. To explore this further, we used MYC plasmids lacking the MB1, MB2, and MB3 domains and confirmed that USP11 influences MYC ubiquitination levels through its interaction with the MB1 domain. Furthermore, we found that DLGAP5, USP11, and MYC form a positive feedback loop that is particularly amplified in GEM-resistant cells. After GEM treatment, increased MYC levels enhance *DLGAP5* transcription, which in turn promotes the binding of USP11 to MYC, stabilizing MYC and resisting the cytotoxic effects of GEM. Disruption of this feedback loop may effectively inhibit the role of MYC in tumor resistance. On the basis of our findings, we propose that DLGAP5 inhibition could serve as an evaluation and adjunctive therapeutic strategy for GEM treatment outcomes.

Limitations of this study still exist. First, we focused on the molecular functions of the DLGAP5-MYC loop in tumor cells without considering the tumor microenvironment. The role of MYC in regulating the immune microenvironment is also significant, and further research could explore the role of DLGAP5 in the tumor immune microenvironment 71-73. Second, the initial drivers of MYC upregulation post-GEM treatment need further investigation, and whether other MYC regulatory mechanisms play essential roles in GEM treatment remains unresolved. Our experiments suggest potential targets to overcome GEM resistance, but further drug research and clinical trials are needed to evaluate their significance.

In conclusion, our study identifies DLGAP5 as a key factor in enhancing GEM resistance in BLCA. We revealed that during GEM chemotherapy, the DLGAP5/USP11/MYC feedback loop promotes glycolysis, thereby contributing to GEM resistance. We hope that, with further pharmacological and clinical research, DLGAP5 may become one of the key factors in overcoming GEM resistance in BLCA patients.

## Materials and Methods

### Human tissue samples

Bladder cancer (BLCA) tissues, including specimens from 53 BLCA patients, were obtained from the Department of Urology at Zhongnan Hospital of Wuhan University (ZNWH). The Institutional Ethics Review Board provided prior authorization for this research (approval number: 2022039K). All participants provided written informed consent, thereby ensuring adherence to ethical standards.

### Cell lines and transfection

The cell cultures used in this study were sourced from the Cell Bank of the Chinese Academy of Sciences in Shanghai, China. T24 cells were cultured in 1640 medium, UM-UC-3 cells were cultured in MEM, and HEK293T cells were cultured in DMEM, with all media enriched with 10% fetal bovine serum. Authentication procedures carried out by the Cell Bank at the Chinese Academy of Sciences (in Shanghai, China) verified that there was no mycoplasma contamination.

Lipofectamine^TM^ 3000 Reagent (L3000015, Invitrogen) was utilized for the transfection of the siRNAs and plasmids, following the instructions provided by the manufacturer. Gene-silencing siRNAs were obtained from GenePharma (Shanghai, China) (Table S3). The Flag-MYC and HA-MYC plasmids were kindly supplied by Prof. Guoliang Qing from Wuhan University, China; the GFP-DLGAP5 and HA-USP11 plasmids were obtained from Miaoling Co., Ltd. (Wuhan, China). Additional plasmids were constructed through molecular cloning techniques and validated via DNA sequencing.

### Chemicals

The chemicals utilized in this research, such as 2-deoxy-D-glucose (2-DG, HY-13966), oxamic acid sodium (HY-W013032A), lactate sodium (HY-B2227B), and pyruvate (HY-Y0781), were obtained from MedChemExpress. Gemcitabine (S1714) and Chloroquine (CQ, S6999) was acquired from Selleck Chemicals. N-Butyl-N-(4-hydroxybutyl) nitrosamine (BBN, B0938) was obtained from TCI Chemicals (Shanghai).

### RNA extraction and quantitative reverse transcription-PCR (qRT-PCR)

RNA was extracted in its entirety via the Magen HiPure Mini Kit (R4111-03) according to the instructions provided by the manufacturer. Bio-Rad iTaq Universal SYBR Green Supermix was used to perform qRT-PCR. The sequences of primers used for qRT-PCR are provided in Table S4.

### Western blot and co-immunoprecipitation (co-IP)

Total protein extraction was achieved via RIPA lysis buffer supplemented with a protease inhibitor cocktail. Proteins were separated by SDS-PAGE and transferred onto PVDF membranes for Western blotting. Following transfer, the membranes were treated with primary and secondary antibodies according to set protocols, and protein signals were identified through chemiluminescence and captured via a BioSpectrum 515 lmaging System (UVP, USA). Table S5 contains information for the antibodies used.

The BeaverBeads^TM^ Protein A/G Immunoprecipitation Kit (22202-100, Beaver) was utilized for co-IP experiments following the supplier's instructions. First, cell lysates were prepared and incubated with primary antibodies overnight at 4°C. Protein A/G magnetic beads were subsequently added and incubated for 3 h at 4°C. After four washes with IP binding buffer, the samples were denatured in 1× loading buffer at 100°C for 10 min. The eluted proteins were analyzed via Western blotting.

### Cell proliferation assay

Cell proliferation was assessed with the MTT assay. A 96-well plate was used to seed cells at a concentration of 3000 cells per well. After treatment, 20 µL of MTT solution (Sigma) was added to each well and incubated for 4 h at 37°C. After the medium was gently removed, 200 µL of dimethyl sulfoxide (DMSO) was added to dissolve the formazan crystals. The mixture was gently shaken to ensure complete dissolution, and the absorbance was measured at 540 nm via a microplate reader.

### Evaluation of cell apoptosis through flow cytometry

Following treatment, the BLCA cells were carefully rinsed with phosphate-buffered saline (PBS), resuspended in 100 µL of 1× Annexin V binding buffer, and then stained with 5 µL each of Annexin V-FITC and propidium iodide (PI) from Sungene Biotech. The staining mixture was incubated in darkness for 30 min. Following the addition of 400 µL of 1× binding buffer, apoptosis was evaluated with a flow cytometer (Beckman, USA).

### Metabolic analysis

BLCA cells (5 × 10^6^) were lysed to extract intracellular lactate, pyruvate, and lactate dehydrogenase (LDH) activity, following protocols provided by Abbkine via the Lactate Assay Kit (KTB1100) and Pyruvate Acid Assay Kit (KTB1121). To analyze glucose uptake, BLCA cells (1 × 10^6^) were seeded in 6-well dishes and cultured in medium without serum for 24 h. The culture supernatant was collected, and glucose levels were quantified via a glucose assay kit (BioVision, #K606-100).

### Immunofluorescence

BLCA cells cultured on coverslips were fixed with 4% paraformaldehyde and treated with 0.4% Triton X-100 for 15 min to permeabilize them. After being blocked with 2% BSA for 30 min, the cells were incubated sequentially with primary antibodies, fluorescent secondary antibodies, and DAPI. Coverslips were mounted with antifade reagent, and imaging was conducted via a confocal microscope (Nikon C2^+^, Japan). The details of the antibodies used are listed in Table S5.

### H&E staining and immunohistochemistry (IHC)

Samples from human and animal tissues were preserved with paraformaldehyde, encased in paraffin, sliced into sections, cleared of paraffin with xylene, and rehydrated through a series of alcohol solutions. For H&E staining, the sections were immersed in 10% hematoxylin solution followed by 1% eosin solution and then coverslipped. For IHC, antigen retrieval was performed via EDTA buffer, followed by serum blocking. The sections were incubated with both primary and secondary antibodies, followed by DAB staining, hematoxylin counterstaining, and then examined under a microscope.

### ^18^F fluoro-D-deoxyglucose (^18^F-FDG) positron emission tomography (PET)-CT imaging

The mechanism of action for ^18^F-FDG PET/CT is based on the Warburg effect, which reflects glucose metabolism levels in the tumor 74. After the mouse subcutaneous BLCA xenograft model was established, the tumor-bearing mice were fasted for 4 h before examination. A mixture of 1.0-1.5% isoflurane and pure air was used to administer anesthesia. The mice were injected with 150 μCi of ^18^F-FDG via the tail vein. Sixty minutes after the injection, imaging was conducted via the InliView-3000B small animal PET/SPECT/CT system (Novel Medical Ltd., Beijing, China).

### Dual-luciferase reporter assay

BLCA cells were cultured in 12-well plates until they reached approximately 70% confluence and then were transfected with specific plasmids for 24 h. Renilla luciferase served as the internal control. The test adhered to the instructions provided by the manufacturer for the Dual-Luciferase® Reporter Assay System (Promega).

### Chromatin immunoprecipitation (ChIP)

ChIP was conducted using T24 cells (1 × 10^7^). First, the cells were fixed with 1% formaldehyde for ten minutes, followed by the addition of 0.125 M glycine to halt the crosslinking process. Following harvest, the cells were broken down on ice for half an hour using ChIP Sonication Cell Lysis Solution, and the chromatin was sheared to break the DNA into pieces. Chromatin fragments were incubated for 8 h at 4°C with either the MYC antibody or the IgG. The protein-DNA mixtures were mixed with Protein A/G magnetic beads and incubated for 3 h. Following washes with both low-salt and high-salt ChIP buffers, the chromatin was eluted and the DNA was subsequently purified. Purified DNA was analyzed via qPCR using primers specific to the DLGAP5 promoter region (details in Table S4).

### GST pull-down assay

A GST pull-down experiment was performed utilizing GST and GST-MYC proteins sourced from CUSABIO Co., Ltd. (Wuhan, China). First, the transfected 293T cells were lysed via IP lysis buffer, and the supernatant was extracted via centrifugation. The lysate was incubated overnight with 2 µg of either GST or GST-MYC protein and then incubated with glutathione magnetic beads for 2 h. The protein complexes were subsequently eluted with 1× SDS buffer and denatured at 100°C for 10 min. The eluted proteins were subjected to Western blot analysis.

### Animal studies

T24 cells were transduced with lentiviruses carrying shNC, shDLGAP5, and MYC constructs (GenePharma, Shanghai) and cultured under puromycin selection (Sigma, 1 µg/mL) to establish stable cell lines (shNC, shDLGAP5, and shDLGAP5+MYC). The Ethics Committee for Animal Welfare of Zhongnan Hospital of Wuhan University granted ethical approval for all the animal studies (approval number: ZN2022109).

For the animal experiments, the mice were randomly divided into groups. Stable T24 cells (1 × 10^7^ cells) were subcutaneously injected into four-week-old male BALB/c nude mice (GemPharmatech Co., Ltd.). Twelve days postinjection, the mice were administered intraperitoneal injections of either PBS or GEM at 25 mg/kg every three days. For drug treatment, no blinding was performed. After 24 days, the mice were imaged via a 5.0 T whole-body magnetic resonance scanning system (uMR Jupiter, United-Imaging Healthcare, China), after which the tumors were surgically removed for further examination. Every six days, the tumor size was measured via the following equation: tumor volume (mm^3^) = (length × width^2^)/2. The mice were randomly allocated to experimental groups, and blinding was not implemented.

The *Dlgap5* knockout mouse (C57BL/6J) was purchased from Cyagen Biosciences (Suzhou, China). Knockout and genotyping strategies are shown in the Figure S5A. Mouse tail DNA was extracted using the TIANamp Genomic DNA Kit (Tiangen Biotech; Beijing; China). PCR was carried out in 25 µL volume for 35 cycles under standard conditions, with all two primers listed above added to each reaction. Primer 1: F1: 5'-ACTTCAGAGGTTGAGCTTGAGTC-3', R1: 5'-GAATGGATCAGTTTGAGCAACTACA-3', Product size: 539 bp; Primer 2: F2: 5'-TGAGACAGAAGATGCCACTGAAG-3', R1: 5'-GAATGGATCAGTTTGAGCAACTACA-3', Product size: 620 bp. Homozygotes: one band with 539 bp; Wildtype allele: one band with 620 bp.

For the BBN-induced BLCA mouse model, 8-week-old male mice were given free access to sterile tap water containing 0.05% BBN for 16 weeks, after which they were replaced with sterile tap water until the end of the experiment. For drug treatment, the mice were administered intraperitoneal injections of either PBS or GEM at 25 mg/kg twice a week for 7 weeks (Figure S5B). All mice were sacrificed at the end of the treatment and the bladder was removed for H&E and IHC staining.

### Bioinformatics processing

For the RNA-seq analysis, we used the “DEseq2” package in R to identify differentially expressed genes. The selection criteria were set at an adjusted *p*-value < 0.05 and |Log2 FC| > 1. For Gene Set Enrichment Analysis (GSEA), the “clusterProfiler” package was utilized. Single-cell RNA sequencing data was analyzed using the “Seurat” package to classify different cell types and examine gene expression matrices.

### Statistical analysis

Statistical analyses were conducted via SPSS 22.0 and GraphPad Prism 9. The results from three independent experiments are presented as the mean ± standard deviation (SD). The statistical significance of the data was evaluated through analysis of variance (ANOVA) and a two-tailed Student's t-test, considering a *p*-value of less than 0.05 as significant.

## Supplementary Material

Supplementary figures and tables.
