# Supplementary material for: DLGAP5 enhances bladder cancer chemoresistance by regulating glycolysis through MYC stabilization
Source: Theranostics. 2025 Jan 20;15(6):2375–92. doi: 10.7150/thno.102730 (PMC11840727; doi:10.7150/thno.102730)
Supplement: Supplementary file 1 — Supplementary figures and tables. [file thnov15p2375s1.pdf]

Supplementary Materials

DLGAP5 enhances bladder cancer chemoresistance by regulating glycolysis through MYC stabilization

Supplementary Figures S1-S16: Pages 2-27

Supplementary Tables S1-S5: Pages 28-33

Supplementary Figures

A

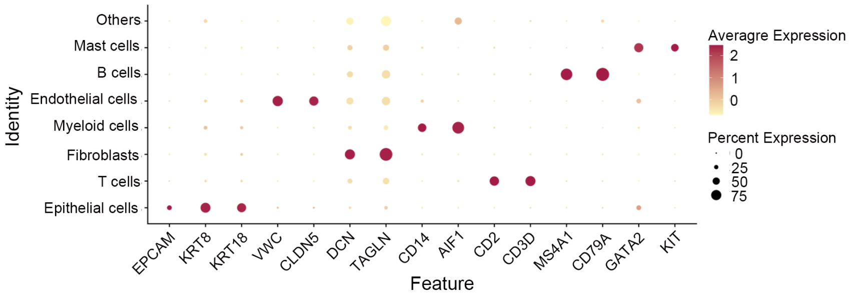

B

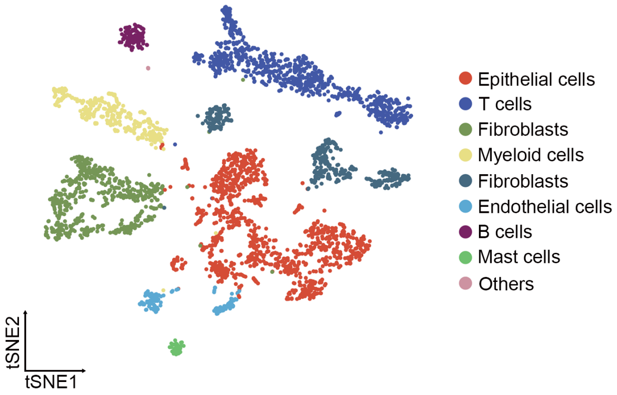

Figure S1. Distribution of DLGAP5 in different cell subsets of bladder cancer (BLCA) cells.

(**A**) The dot plot showing the marker genes of each cell type in tumors from MIBC patient. (**B**) t-SNE map of single-cell RNA-seq analyses of chemosensitive and chemoresistant tumors from MIBC patients, colored by cell subtypes.

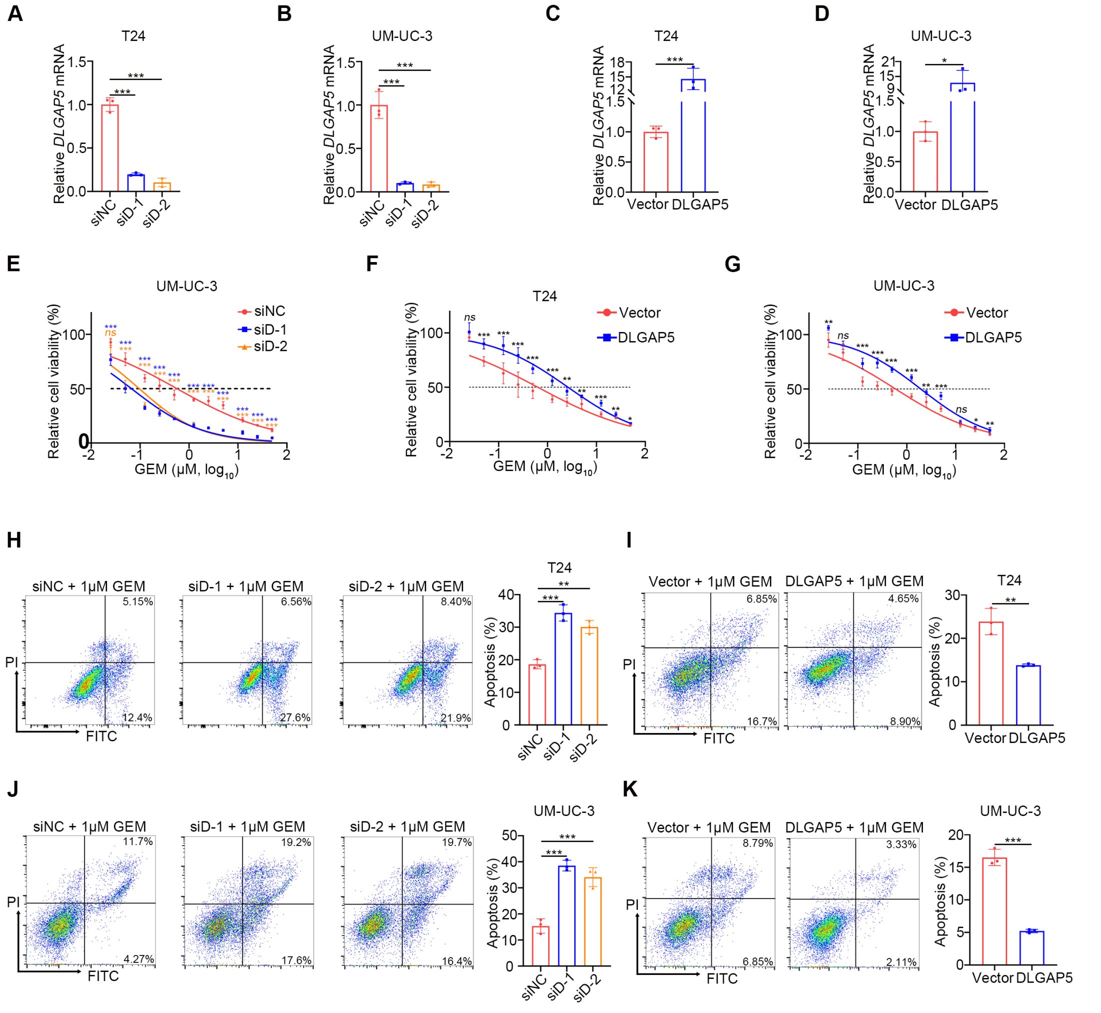

Figure S2. DLGAP5 enhances GEM resistance in BLCA cells.

Quantification of *DLGAP5* mRNA expression following *DLGAP5* knockdown in T24 (**A**) and UM-UC-3 (**B**) cells by qRT-PCR (n = 3). Quantification of *DLGAP5* mRNA expression following DLGAP5 overexpression in T24 (**C**) and UM-UC-3 (**D**) cells by qRT-PCR (n = 3). (**E**) Cell viability of UM-UC-3 cells with *DLGAP5* knockdown after treatment with various concentrations of GEM for 48 h, as measured via MTT assay (n = 6). Cell viability of T24 (**F**) and UM-UC-3 (**G**) cells with DLGAP5 overexpression after 48 h GEM treatment at various concentrations, tested by MTT assay (n = 6). Analysis of apoptosis in T24 cells with either *DLGAP5* knockdown (**H**) or DLGAP5 overexpression (**I**) following 1 μM GEM treatment for 48 h (n = 3). Apoptosis in UM-UC-3 cells with either *DLGAP5* knockdown (**J**) or DLGAP5 overexpression (**K**) after 48 h of 1 μM GEM treatment (n = 3). Statistical significance of data was ascertained by two-tailed unpaired Student's t-test (C, D, F, G, I, K) and one-way ANOVA with Tukey's multiple comparisons test analyses (A, B, E, H, J). All statistical data are presented as mean ± SD, * $p < 0.05$, ** $p < 0.01$, *** $p < 0.001$.

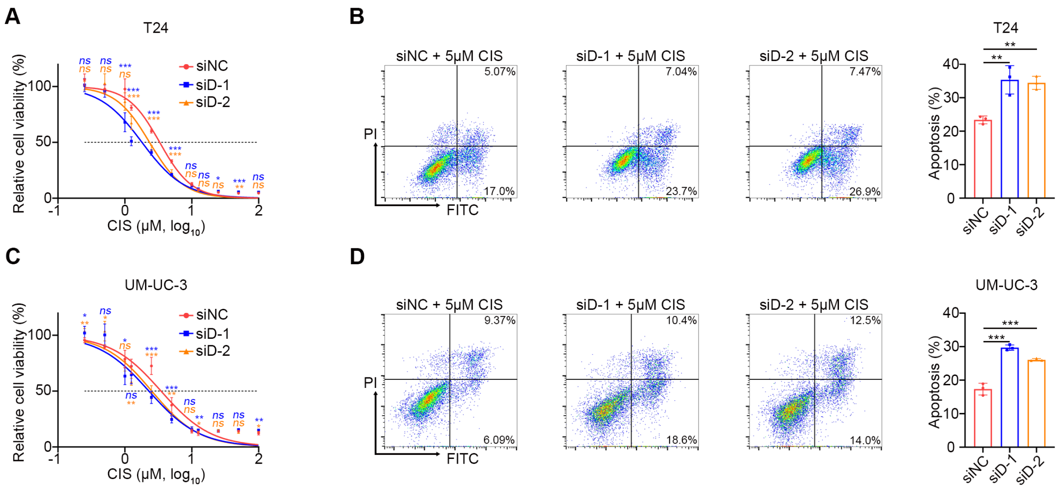

Figure S3. DLGAP5 enhances GEM resistance in BLCA cells.

(**A**) Cell viability of T24 cells with *DLGAP5* knockdown after 48 h of treatment with various concentrations of CIS, as measured via MTT assay (n = 6). (**B**) Analysis of apoptosis in T24 cells with DLGAP5 overexpression following 5 μM CIS treatment for 48 h (n = 3). (**C**) Cell viability of UM-UC-3 cells with *DLGAP5* knockdown after 48 h of treatment with various concentrations of CIS, as measured via MTT assay (n = 6). (**D**) Analysis of apoptosis in UM-UC-3 cells with DLGAP5 overexpression following 5 μM CIS treatment for 48 h (n = 3). Statistical significance of data was ascertained by one-way ANOVA with Tukey's multiple comparisons test analyses (A-D). All statistical data are presented as mean ± SD, * $p < 0.05$, ** $p < 0.01$, *** $p < 0.001$.

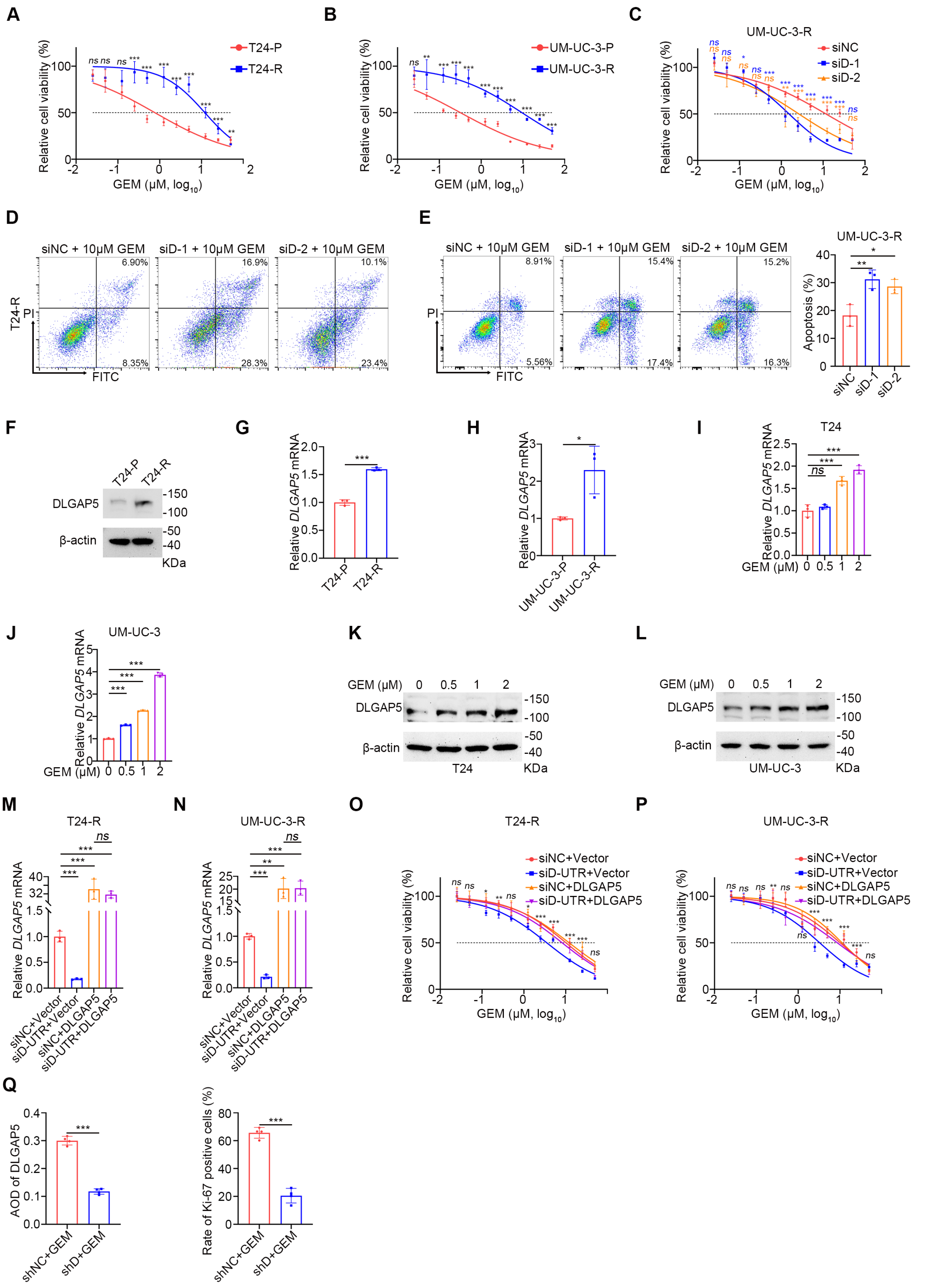

Figure S4. DLGAP5 enhances GEM resistance in BLCA cells.

Cell viability of T24-P and T24-R cells (**A**), UM-UC-3-P and UM-UC-3-R cells (**B**) after 48 h of treatment with different concentrations of GEM, as measured via MTT assay (n = 6). (**C**) Cell viability of UM-UC-3-R cells with *DLGAP5* knockdown after 48 h of treatment with various concentrations of GEM, as measured via MTT assay (n

= 6). (**D**) Apoptotic cells of T24-R cells with *DLGAP5* knockdown after 48 h of 10 μM GEM treatment (*n* = 3). (**E**) Apoptosis analysis of UM-UC-3-R cells with *DLGAP5* knockdown after 48 h of 10 μM GEM treatment (*n* = 3). (**F**) Western blot analysis of DLGAP5 proteins in T24-P and T24-R cells. Quantification of *DLGAP5* mRNA expression in T24-P and T24-R cells (**G**) and UM-UC-3-P and UM-UC-3-R cells (**H**) via qRT-PCR (*n* = 3). Quantification of *DLGAP5* mRNA expression in T24 (**I**) and UM-UC-3 cells (**J**) after 24 h GEM treatment at various concentrations via qRT-PCR (*n* = 3). Western blot analysis of DLGAP5 proteins in T24 (**K**) and UM-UC-3 (**L**) cells after 24 h of treatment with different concentrations of GEM. Quantification of *DLGAP5* mRNA expression in T24-R (**M**) and UM-UC-3-R (**N**) cells re-expressing DLGAP5 after knocking down *DLGAP5* by siRNAs targeting the 3'UTR of DLGAP5. (n = 3). Viability of T24-R (**O**) and UM-UC-3-R (**P**) cells with indicated treatment after 48 h GEM treatment at various concentrations, determined using the MTT assay (*n* = 6). The asterisk indicates statistical significance between siD-UTR+Vector and siD-UTR+DLGAP5. (**Q**) Statistical average optical density (AOD) value of DLGAP5 and the rate of Ki-67 positive cells from IHC staining analysis in Figure 1L. Statistical significance of data was ascertained by two-tailed unpaired Student's t-test (A, B, G, H, Q) and one-way ANOVA with Tukey's multiple comparisons test analyses (C, E, I, J, M-P). All statistical data are presented as mean ± SD, * $p < 0.05$, ** $p < 0.01$, *** $p < 0.001$.

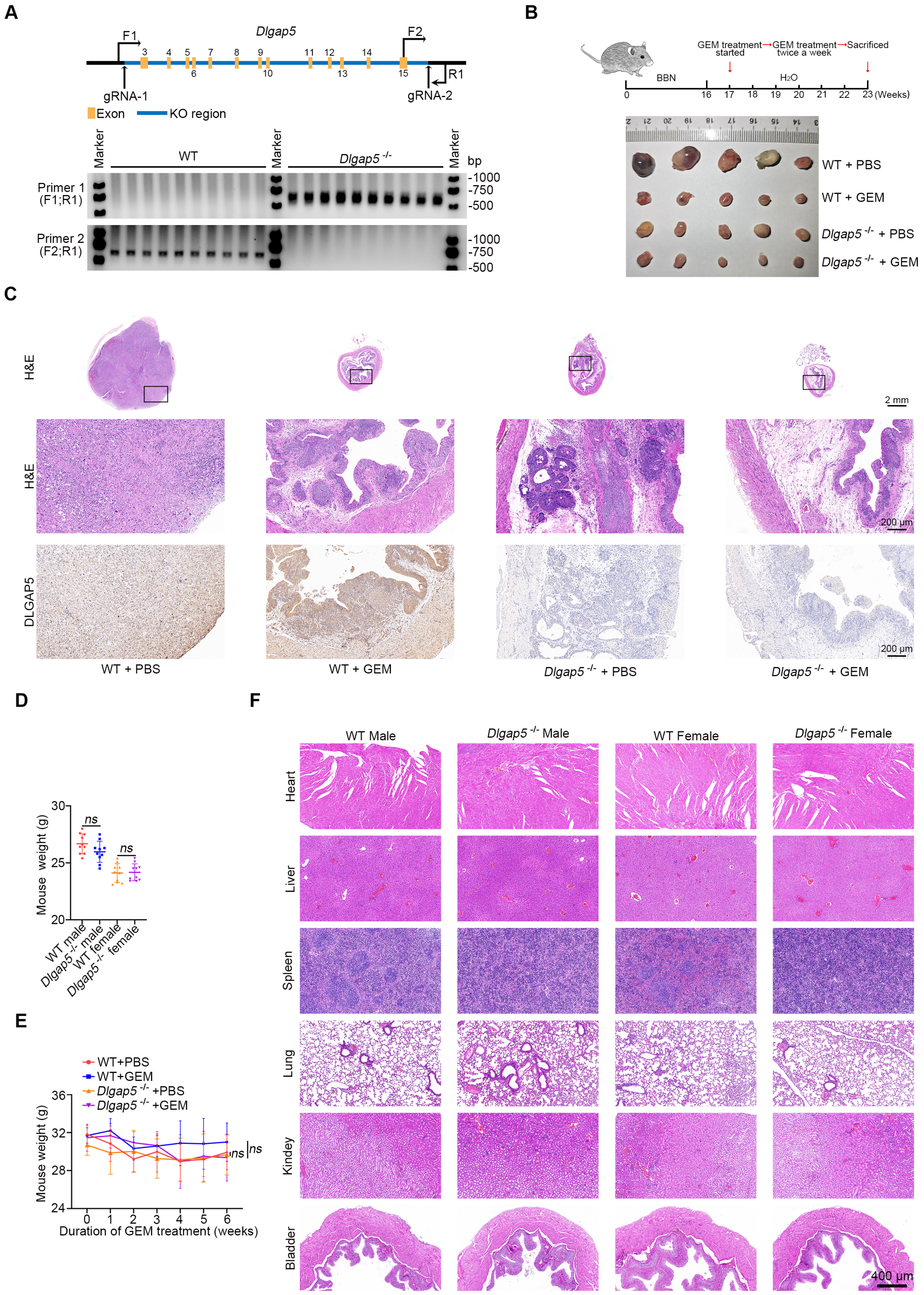

Figure S5. DLGAP5 enhances GEM resistance in a BBN-induced spontaneous model of BLCA.

(**A**) Knockout strategies (top) and genotyping results of WT and *Dlgap5*$^{-/-}$ mice. *Dlgap5*$^{-/-}$: one band with 539 bp; WT: one band with 620 bp. (**B**) *In vivo* BBN-induced spontaneous model construction and drug treatment (top). General view of dissected

bladder from each group (bottom, $n = 5$). (**C**) Representative H&E and DLGAP5 IHC staining analysis of bladder from the BBN-induced spontaneous model. (**D**) 6-week-old body weight of mice in each group ($n = 10$). (**E**) Body weight of each group during GEM treatment ($n = 5$). (**F**) Representative H&E staining imaging of the heart, liver, lung, spleen, kidney, and bladder from each group. Scale bar, 500 μm. Statistical significance of data was ascertained by one-way ANOVA with Tukey's multiple comparisons test analyses (D, E). All statistical data are presented as mean ± SD, * $p < 0.05$, ** $p < 0.01$, *** $p < 0.001$.

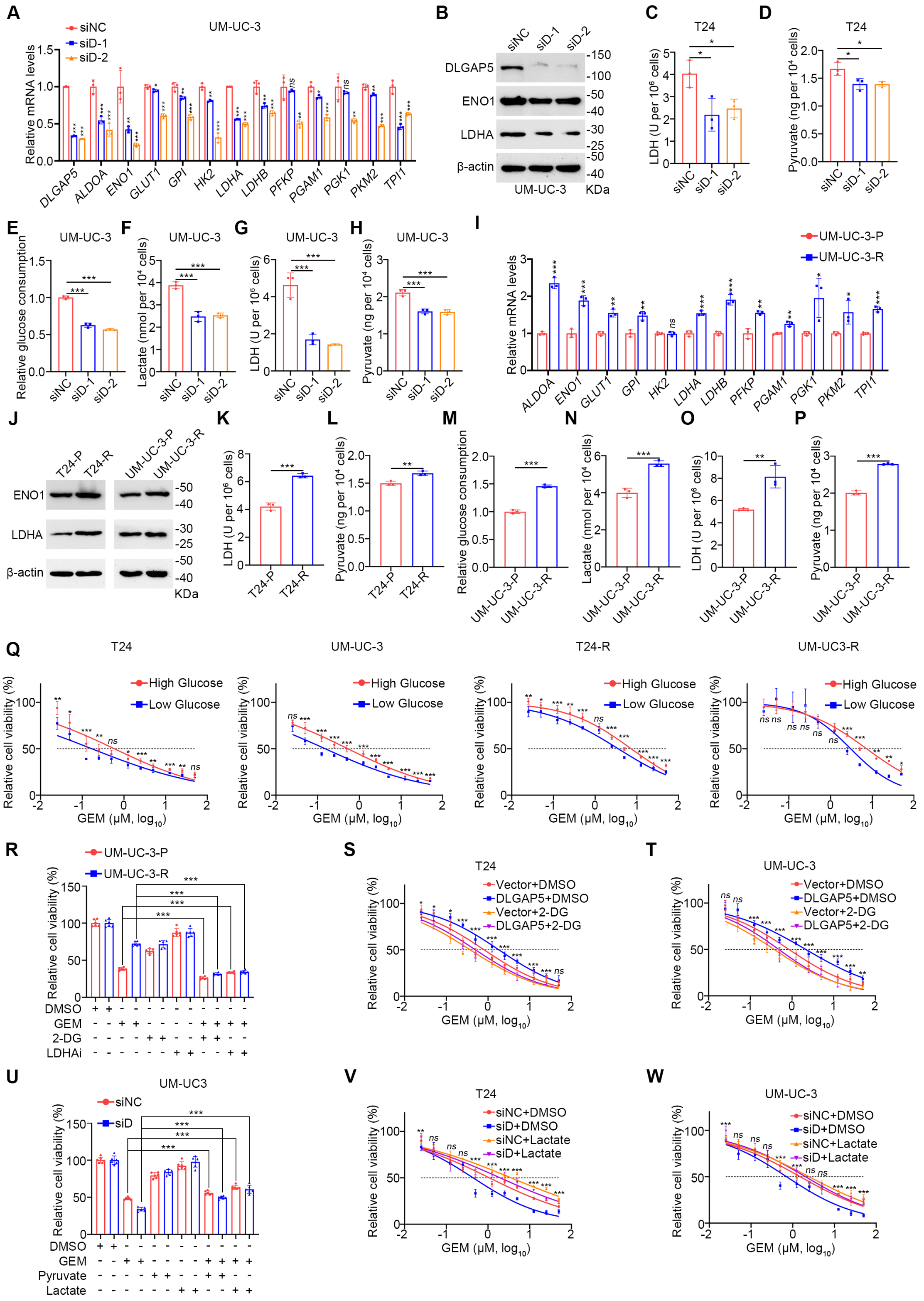

Figure S6. DLGAP5 influences GEM resistance in BLCA by regulating glycolysis. (**A**) Transcriptional changes in glycolysis-related genes in UM-UC-3 cells following *DLGAP5* knockdown (n = 3). (**B**) Western blot analysis of knockdown *DLGAP5* on ENO1 and LDHA proteins in UM-UC-3 cells. The relative intracellular LDH activity (**C**) and pyruvate concentration (**D**) in T24 cells after knockdown *DLGAP5* (n = 3). The

relative glucose uptake (**E**), intracellular lactate production (**F**), intracellular LDH activity (**G**), pyruvate concentration (**H**) in UM-UC-3 cells after knockdown *DLGAP5* ($n = 3$). (**I**) mRNA levels of glycolysis-related genes in UM-UC-3-P and UM-UC-3-R cells ($n = 3$). (**J**) Protein levels of ENO1 and LDHA in T24-P, T24-R cells, and UM-UC-3-P, UM-UC-3-R cells. The relative intracellular LDH activity (**K**) and pyruvate concentration (**L**) in T24-P and T24-R cells. The relative glucose uptake (**M**), intracellular lactate production (**N**), intracellular LDH activity (**O**), pyruvate concentration (**P**) in UM-UCC-3-P and UM-UC-3-R cells after knockdown *DLGAP5*. (**Q**) Cell viability of T24, UM-UC-3, T24-R, UM-UC-3-R cells with low (1500 mg/L) or high (4500 mg/L) levels glucose medium after 48 h GEM treatment at various concentrations, determined using the MTT assay ($n = 6$). (**R**) UM-UC-3-P and UM-UC-3-R cells were treated with the indicated combinations of GEM (10 μM), 2-DG (2 mM), and oxamate (10 mM) before measuring cell viability at 48 h ($n = 6$). Viability of T24 (**S**) and UM-UC-3 (**T**) cells with specified treatment after treated with the indicated combinations of concentration-gradient GEM and 2-DG (2 mM) was measured at 48 h ($n = 6$). The asterisk indicates statistical significance between DLGAP5+DMSO and DLGAP5+2-DG. (**U**) siNC and siDLGAP5 UM-UC-3 cells were treated with the indicated combinations of GEM (1 μM), pyruvate (2 mM), and lactate (10 mM) before measuring cell viability at 48 h ($n = 6$). Viability of T24 (**V**) and UM-UC-3 (**W**) cells with specified treatment after treated with the indicated combinations of concentration-gradient GEM and Lactate (10 mM) was measured at 48 h ($n = 6$). The asterisk indicates statistical significance between siD+DMSO and siD+Lactate. Statistical significance of data was ascertained by two-tailed unpaired Student's t-test (I, K-Q, R, U) and one-way ANOVA with Tukey's multiple comparisons test analyses (A, C-H, S, T, V, W). All statistical data are presented as mean ± SD, * $p < 0.05$, ** $p < 0.01$, *** $p < 0.001$.

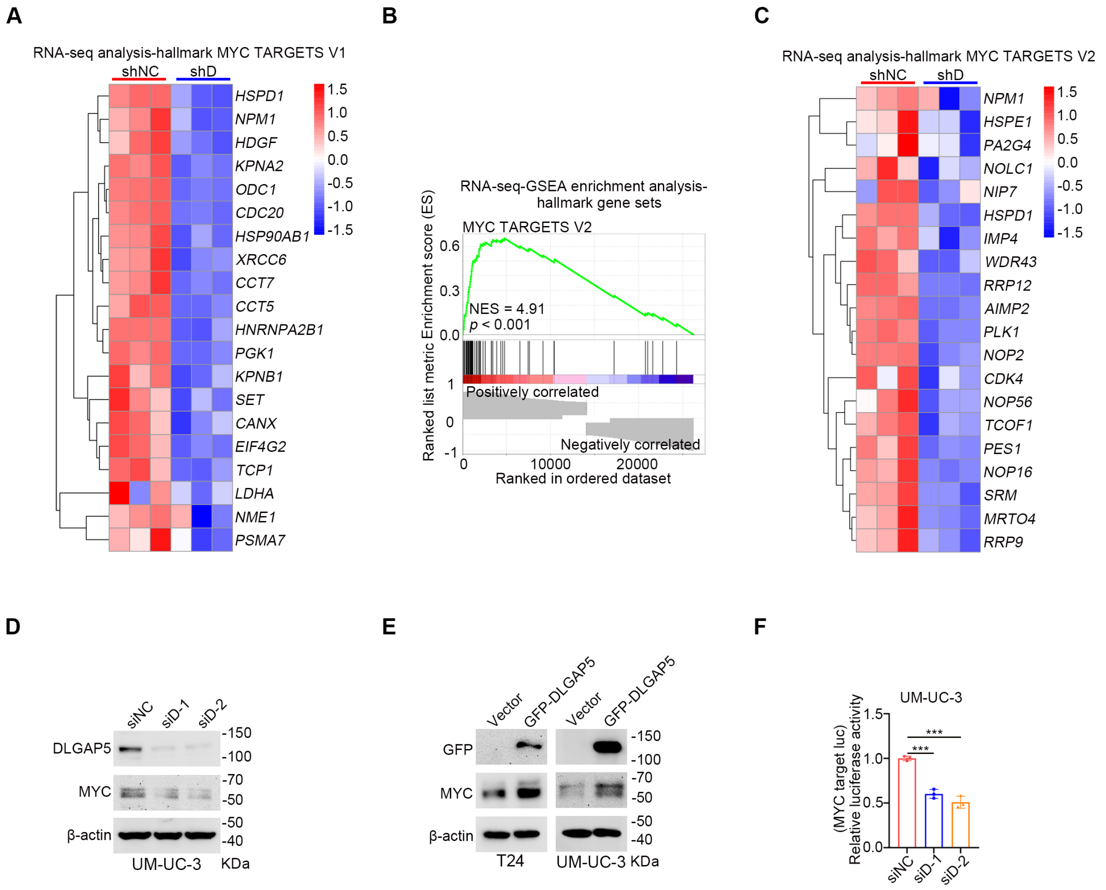

Figure S7. The role of MYC in DLGAP5-mediated GEM resistance.

(**A**) Heatmap analysis of genes with significant changes in the hallmark MYC TARGET V1 gene set upon *DLGAP5* knockdown in T24 cells (n = 3). (**B**) GSEA of *DLGAP5* knockdown in the hallmark MYC TARGET V2 gene set. (**C**) Heatmap analysis of genes with significant changes in the hallmark MYC TARGET V2 gene set upon *DLGAP5* knockdown in T24 cells (n = 3). (**D**) Western blot analysis of knockdown *DLGAP5* on MYC proteins in UM-UC-3 cells. (**E**) Western blot analysis of overexpression DLGAP5 on MYC proteins in T24 and UM-UC-3 cells. (**F**) UM-UC-3 cells were transfected with siDLGAP5 for 24 h, transfected with 5× E-box luciferase reporter for 48 h, and finally subjected to a dual-luciferase reporter assay (n = 3). Statistical significance of data was ascertained by one-way ANOVA with Tukey's multiple comparisons test analyses (F). All statistical data are presented as mean ± SD, * p < 0.05, ** p < 0.01, *** p < 0.001.

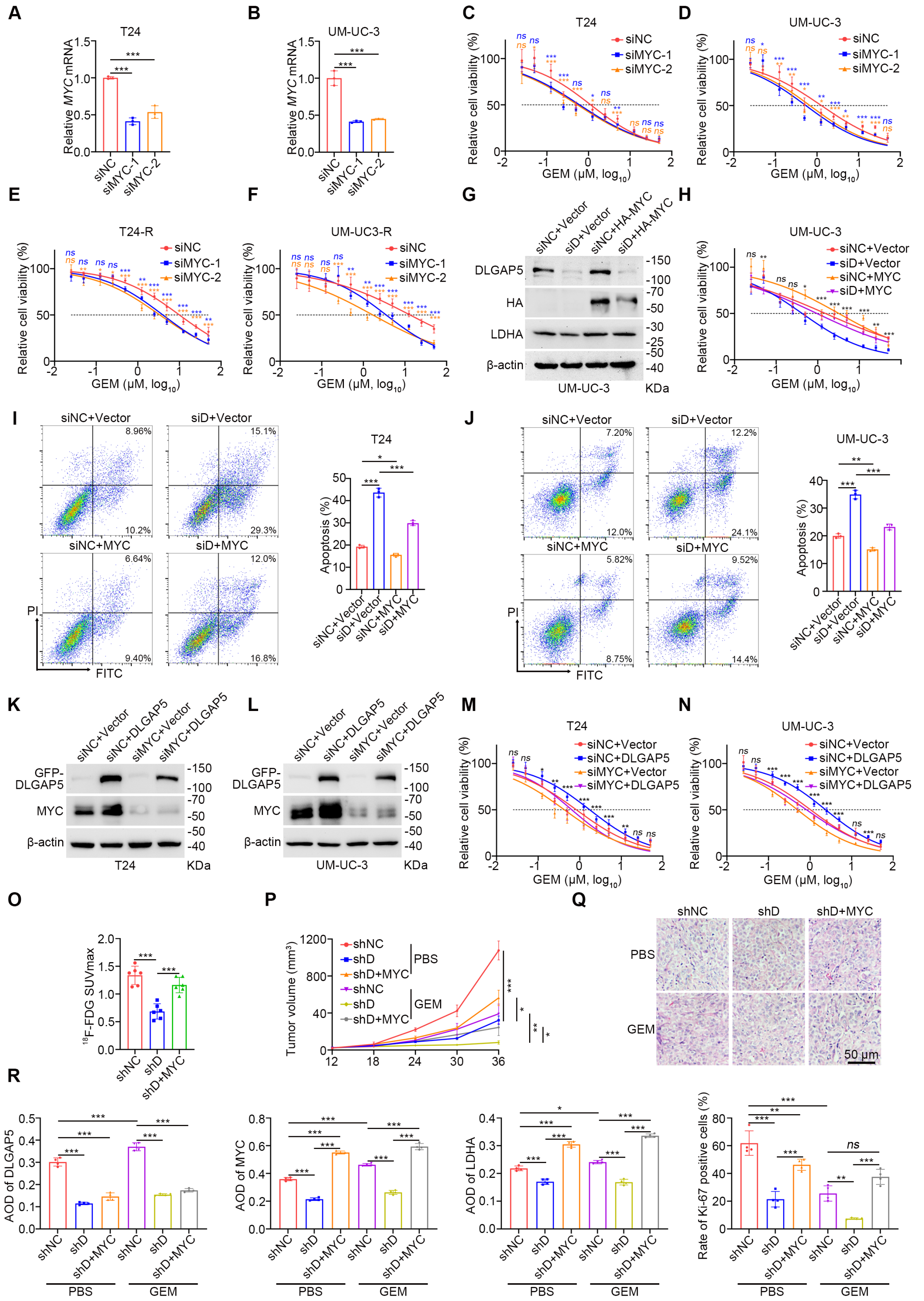

Figure S8. The role of MYC in DLGAP5-mediated GEM resistance.

After knocking down *MYC* in T24 (**A**) and UM-UC-3 (**B**) cells, the mRNA level of *MYC* was detected via qRT-PCR ($n = 3$). Cell viability of T24 (**C**), UM-UC-3 (**D**), T24-R (**E**), UM-UC-3-R (**F**) cells with *MYC* knockdown after 48 h GEM treatment at various concentrations, determined using the MTT assay ($n = 6$). (**G**) Western blots showing

the expression of DLGAP5, HA-MYC, and LDHA proteins in UM-UC-3 cells after *DLGAP5* knockdown and MYC overexpression. (**H**) Viability of UM-UC-3 cells with indicated treatment after 48 h GEM treatment at various concentrations, determined using the MTT assay (n = 6). The asterisk indicates statistical significance between siD+Vector and siD+MYC. Apoptosis analysis of T24 (**I**) and UM-UC-3 (**J**) cells with indicated treatment after 48 h of 1 μM GEM treatment (n = 3). Western blots showing the expression of GFP-DLGAP5 and MYC proteins in T24 (**K**) and UM-UC-3 (**L**) cells after DLGAP5 overexpression and *MYC* knockdown. Viability of T24 (**M**) and UM-UC-3 (**N**) cells with indicated treatment after 48 h GEM treatment at various concentrations, determined using the MTT assay (n = 6). The asterisk indicates statistical significance between siNC+DLGAP5 and siMYC+DLGAP5. (**O**) Statistical values of SUVmax were analyzed via ^{18}F-FDG PET-CT imaging (n = 6). (**P**) Tumor growth of the indicated grafted mice treated with GEM was measured (n = 3). (**Q**) Representative H&E staining analysis of subcutaneous tumor tissues in xenograft models. The scale bar is 50 μm. (**R**) Statistical AOD value for DLGAP5, MYC, LDHA and the rate of Ki-67 positive cells from IHC staining analysis in Figure 3J. Statistical significance of data was ascertained by one-way ANOVA with Tukey's multiple comparisons test analyses (A-F, H-J, M-P, R). All statistical data are presented as mean ± SD, * $p < 0.05$, ** $p < 0.01$, *** $p < 0.001$.

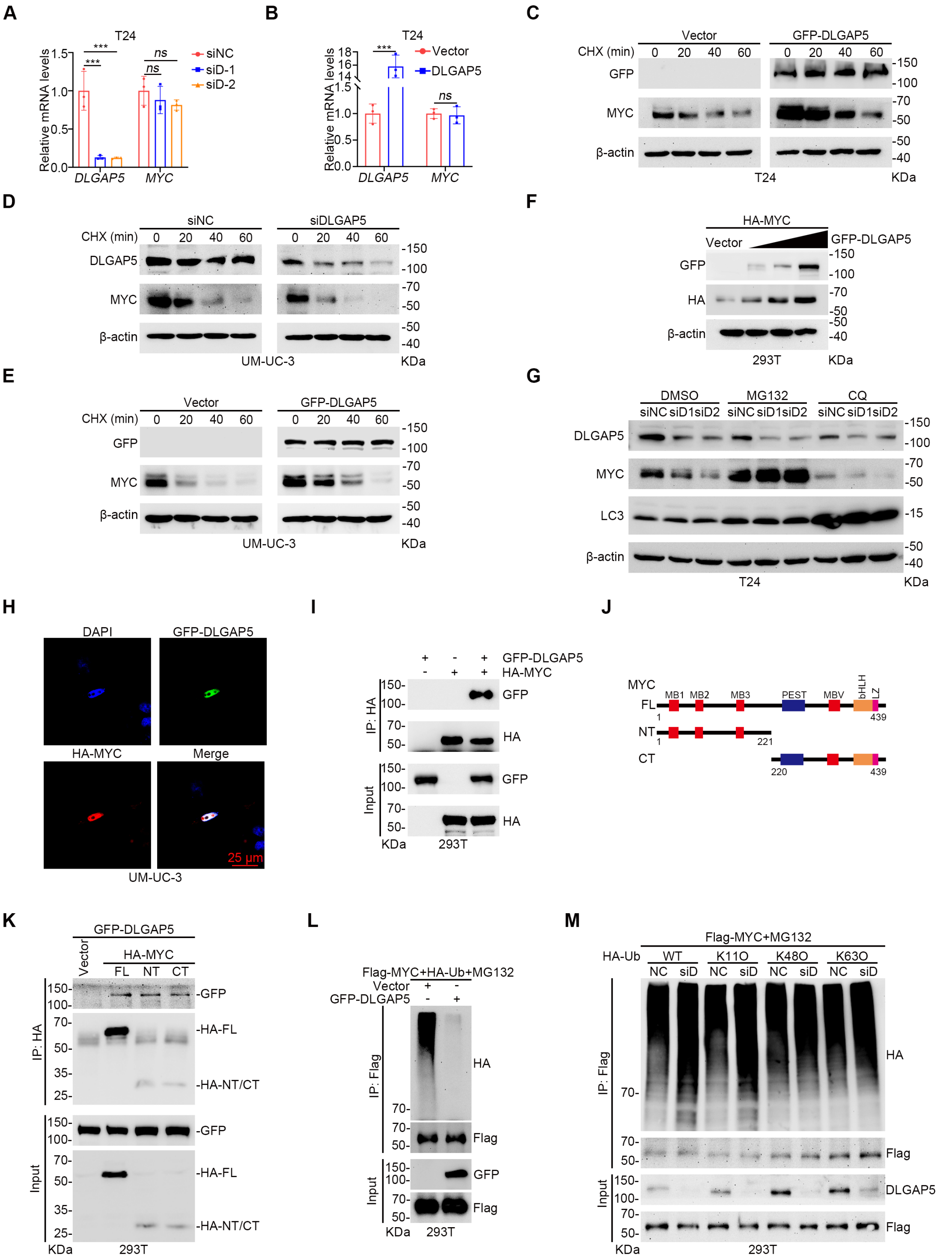

Figure S9. DLGAP5 stabilizes MYC.

After knocking down (**A**) and overexpressing (**B**) DLGAP5 in T24 cells, the mRNA level was detected via qRT-PCR (n = 3). (**C**) Western blots of the effect of overexpressing DLGAP5 on MYC degradation in T24 cells incubated with CHX (50 μg/mL) for the indicated time points. Western blot analysis of the effect of knockdown (**D**) and overexpressing (**E**) DLGAP5 on MYC degradation in UM-UC-3 cells

incubated with CHX (50 μg/mL) for the indicated time points. (**F**) 293T cells were transfected with the described plasmids for 48 h and detected via subsequent Western blots. (**G**) T24 cells were transfected with siNC or siDLGAP5 for 48 h and then treated with DMSO or MG132 (10 μM) or CQ (100 μM) for 8 h before lysis. Protein levels were analyzed via Western blot. (**H**) Confocal imaging confirming that DLGAP5 co-localized with MYC in the nucleus of UM-UC-3 cells. The scale bar is 25 μm. (**I**) Co-IP assay showing that exogenous DLGAP5 interacts with MYC in 293T cells. (**J**) Schematic representation of various MYC truncations. (**K**) Co-IP assay showed that DLGAP5-NT interacts with MYC-NT and MYC-CT in 293T cells. (**L**) 293T cells were transfected with the specified plasmids for 48 h, followed via an 8 h treatment with 10 μM MG132. Western blots indicated exogenous ubiquitination of MYC after DLGAP5 overexpression. (**M**) 293T cells were transfected with the specified plasmids for 48 h, followed via an 8 h treatment with 10 μM MG132. Ubiquitination assays were conducted to examine the specific ubiquitin chain linkage catalyzed via DLGAP5 on MYC proteins. Statistical significance of data was ascertained by two-tailed unpaired Student's t-test (B) and one-way ANOVA with Tukey's multiple comparisons test analyses (A). All statistical data are presented as mean ± SD, * $p < 0.05$, ** $p < 0.01$, *** $p < 0.001$.

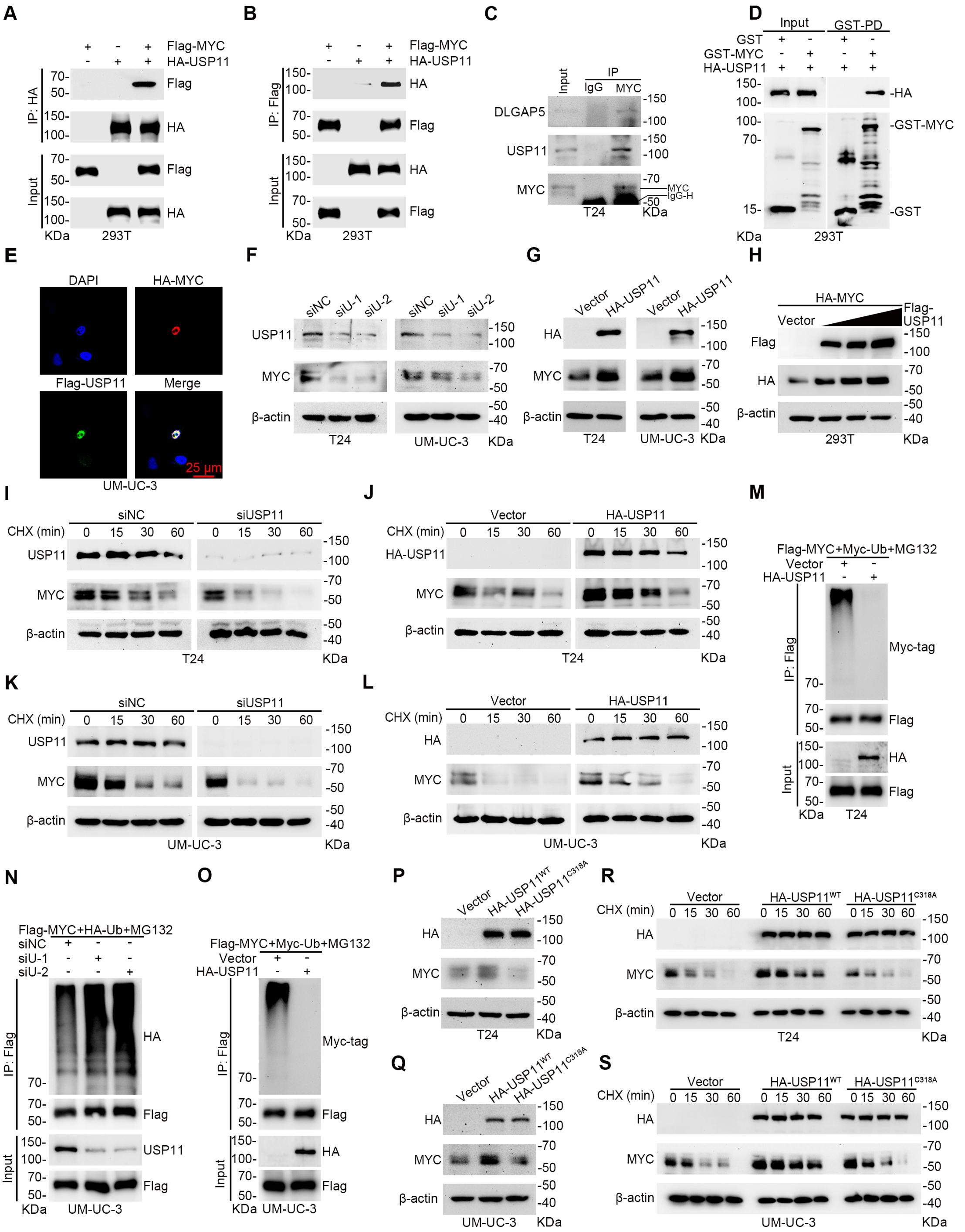

Figure S10. The deubiquitinating enzyme USP11 regulates MYC stability.

(**A-B**) Co-IP assay showing that exogenous USP11 interacts with MYC in 293T cells. (**C**) Co-IP assay showing that endogenous DLGAP5 and USP11 interacts with MYC in T24 cells. (**D**) 293T cells were transfected with HA-USP11 for 48 h and then GST pulldown assay showed that USP11 interacts with MYC *in vitro*. (**E**) Confocal imaging confirming that USP11 co-localized with MYC in the nucleus of UM-UC-3 cells. The scale bar is 25 μm. (**F**) Western blot analysis of knockdown *USP11* on MYC proteins

in T24 and UM-UC-3 cells. (**G**) Western blot analysis of overexpressing USP11 on MYC proteins in T24 and UM-UC-3 cells. (**H**) 293T cells were transfected with the described plasmids for 48 h and detected via subsequent Western blots. Western blot analysis of the effect of and knockdown (**I**) and overexpressing (**J**) USP11 on MYC degradation in T24 cells incubated with CHX (50 μg/mL) for the indicated time points. Western blot analysis of the effect of and knockdown (**K**) and overexpressing (**L**) USP11 on MYC degradation in UM-UC-3 cells incubated with CHX (50 μg/mL) for the indicated time points. (**M**) T24 cells were transfected with the specified plasmids for 48 h, followed via an 8 h treatment with 10 μM MG132. To demonstrate the effect of knockdown (**N**) or overexpression (**O**) of USP11 on MYC ubiquitination, UM-UC-3 cells were transfected with the specified plasmids for 48 h, followed via an 8 h treatment with 10 μM MG132. Western blots showed exogenous ubiquitination of MYC after USP11 overexpression. 293T cells were transfected with the specified plasmids for 48 h, Western blot analysis MYC proteins in T24 (**P**) and UM-UC-3 (**Q**) cells. Western blot analysis of MYC half-life after USP11 (WT) and USP11 (C318A) were overexpressed in T24 (**R**) and UM-UC-3 (**S**) cells. The cells were incubated with CHX (50 μg/mL) for the indicated times.

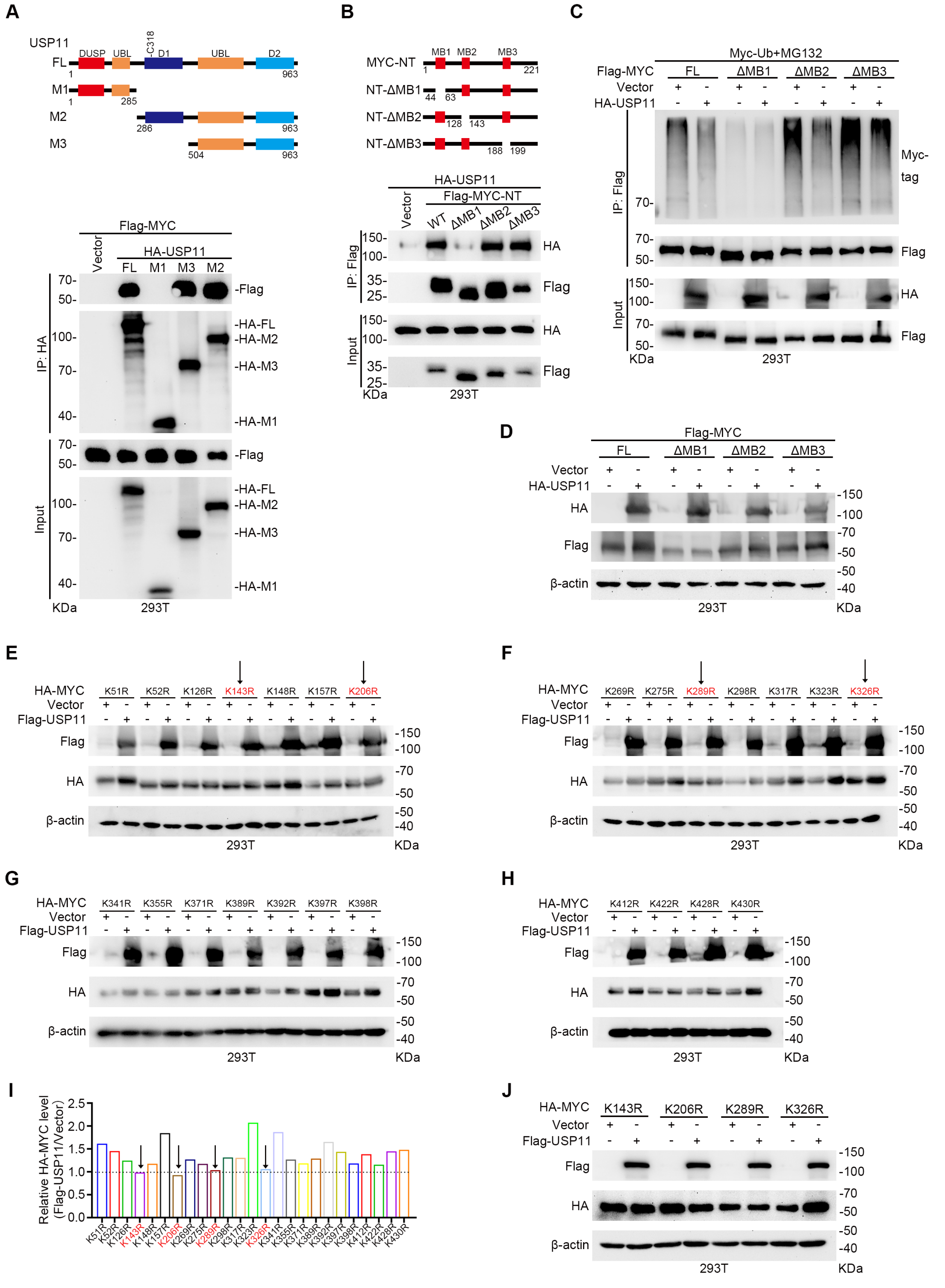

Figure S11. The deubiquitinating enzyme USP11 regulates MYC stability.

(**A**) Schematic representation of various USP11 truncations (top) and co-IP assay showing that USP11-M3 domain interacts with MYC in 293T cells (bottom). (**B**) Schematic representation of various MYC-NT deletion mutations (top) and co-IP assay showing that USP11 interacts with MYC-NT MB1 domain in 293T cells (bottom). (**C**) 293T cells were transfected with the specified plasmids for 48 h, followed via an 8 h

treatment with 10 μM MG132. The ubiquitination assay investigates USP11's ability to regulate the ubiquitination of MYC deletion mutations. (**D**) Western blot analysis demonstrating the regulation of MYC deletion mutations via overexpressed USP11. (**E-I**) 25 HA-tagged MYC mutants were co-transfected with Flag-USP11 into 293T cells. Expression levels of MYC mutants were detected using Western blots. Protein levels were quantified based on the Western blot analysis results (I). (**J**) USP11 with indicated HA-MYC lysine residue mutants were transfected into 293T cells for 48 h. Western blots analysis evaluating protein expression.

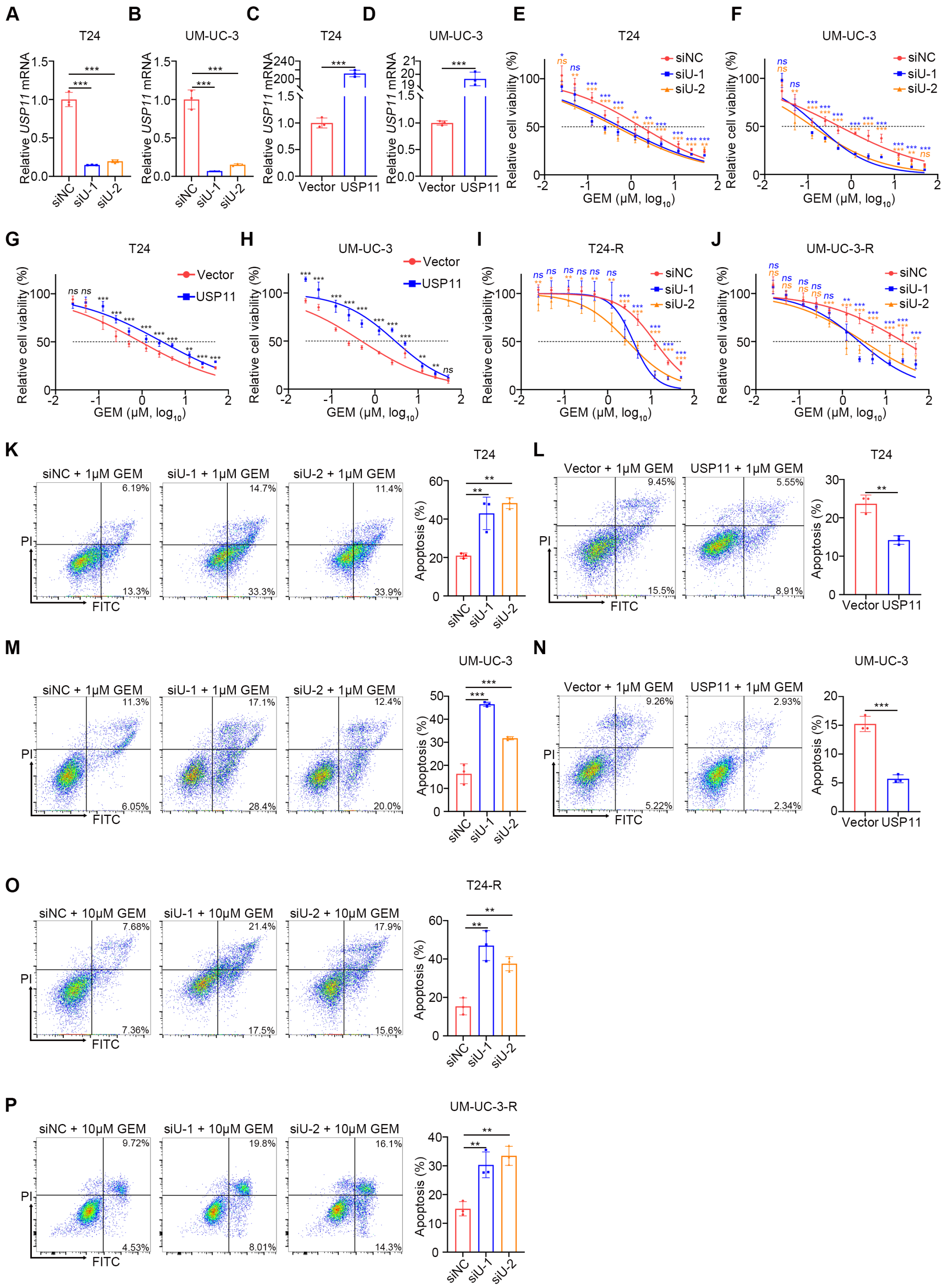

Figure S12. USP11 promotes GEM resistance.

Quantification of *USP11* mRNA expression following *USP11* knockdown in T24 (**A**) and UM-UC-3 (**B**) cells via qRT-PCR (n = 3). Quantification of *USP11* mRNA expression following USP11 overexpression in T24 (**C**) and UM-UC-3 (**D**) cells via qRT-PCR (n = 3). Cell viability of T24 (**E**) and UM-UC-3 (**F**) cells with *USP11* knockdown after 48 h GEM treatment at various concentrations, determined using the

MTT assay (*n* = 6). Cell viability of T24 (**G**) and UM-UC-3 (**H**) cells with USP11 overexpression after 48 h GEM treatment at various concentrations, determined using the MTT assay (*n* = 6). Cell viability of T24-R (**I**) and UM-UC-3-R (**J**) cells with *USP11* knockdown after 48 h GEM treatment at various concentrations, determined using the MTT assay (*n* = 6). Apoptosis in T24 cells with either USP11 knockdown (**K**) or overexpression (**L**) after 48 h of 1 μM GEM treatment (*n* = 3). Apoptosis in UM-UC-3 cells with either *USP11* knockdown (**M**) or USP11 overexpression (**N**) after 48 h of 1 μM GEM treatment (*n* = 3). Apoptosis in T24-R (**O**) and UM-UC-3-R (**P**) cells with *DLGAP5* knockdown after 48 h of 10 μM GEM treatment (*n* = 3). Statistical significance of data was ascertained by two-tailed unpaired Student's t-test (C, D, G, H, L, N) and one-way ANOVA with Tukey's multiple comparisons test analyses (A, B, E, F, I, J, K, M, O, P). All statistical data are presented as mean ± SD, * $p < 0.05$, ** $p < 0.01$, *** $p < 0.001$.

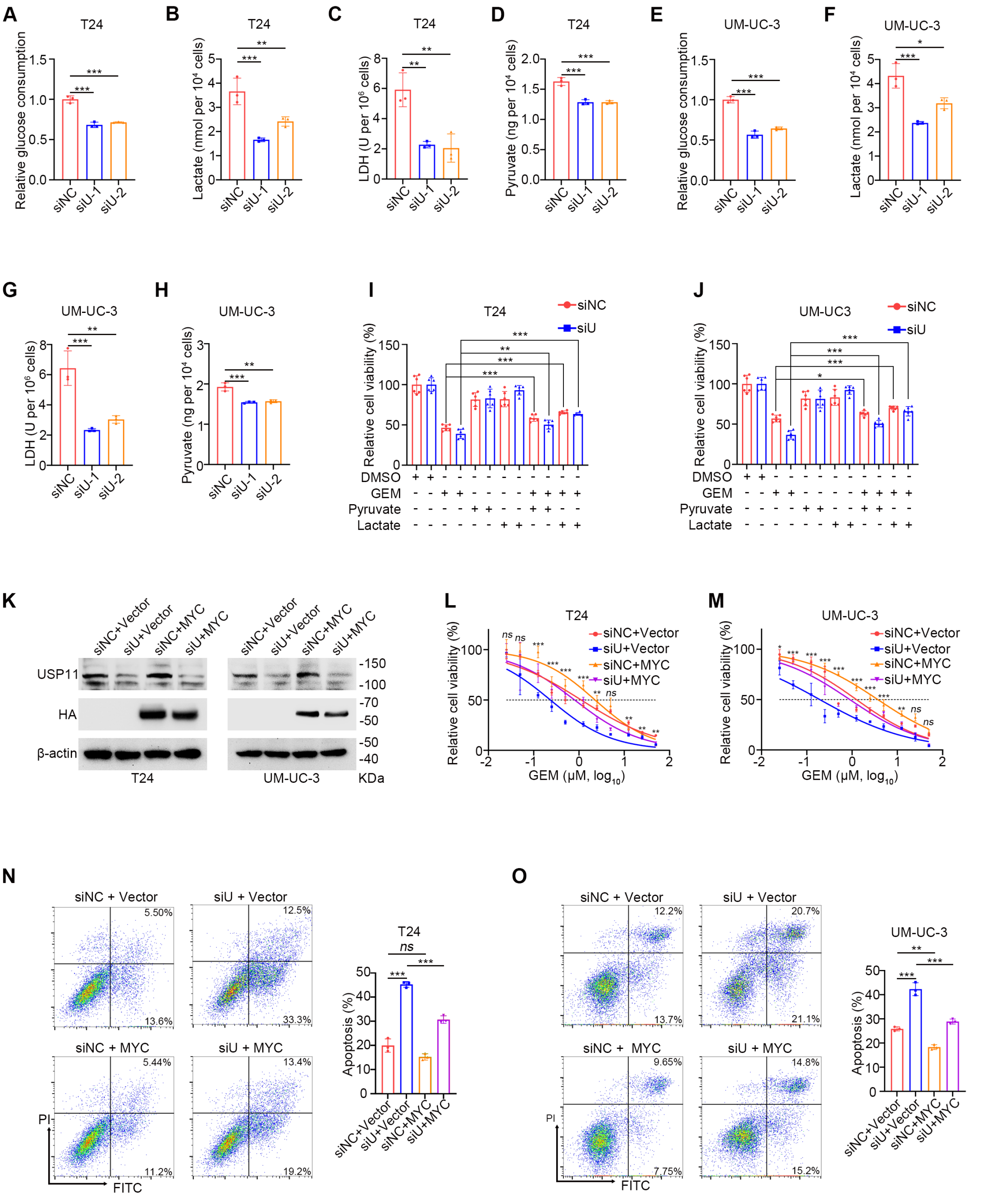

Figure S13. The deubiquitinating enzyme USP11 regulates MYC stability and promotes GEM resistance.

The relative glucose uptake (**A**), intracellular lactate production (**B**), intracellular LDH activity (**C**), pyruvate concentration (**D**) in T24 cells after knockdown *USP11* (n = 3). The relative glucose uptake (**E**), intracellular lactate production (**F**), intracellular LDH activity (**G**), pyruvate concentration (**H**) in T24 cells after knockdown *USP11* (n = 3). siNC and siUSP11 T24 (**I**) and UM-UC-3 (**J**) cells were treated with the indicated combinations of GEM (1 μM), pyruvate (2 mM), and lactate (10 mM) before measuring cell viability at 48 h (n = 6). (**K**) Western blots showing the expression of USP11, HA-

MYC proteins in T24 and UM-UC-3 cells after *USP11* knockdown and MYC overexpression. Viability of T24 (**L**) and UM-UC-3 (**M**) cells with indicated treatment after 48 h GEM treatment at various concentrations, determined using the MTT assay ($n = 6$). The asterisk indicates statistical significance between siU+Vector and siU+MYC. Apoptosis analysis of T24 (**N**) and UM-UC-3 (**O**) cells with indicated treatment after 48 h of 1 μM GEM treatment ($n = 3$). Statistical significance of data was ascertained by two-tailed unpaired Student's t-test (I, J) and one-way ANOVA with Tukey's multiple comparisons test analyses (A-H, L-O). All statistical data are presented as mean ± SD, * $p < 0.05$, ** $p < 0.01$, *** $p < 0.001$.

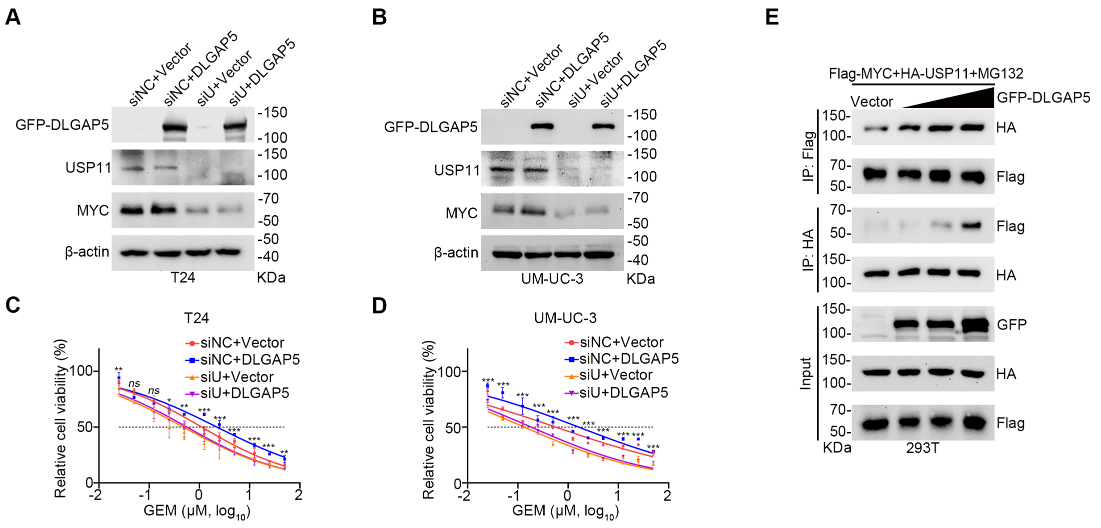

Figure S14. The DLGAP5-USP11-MYC feedback loop induces GEM resistance in BLCA cells.

Western blots showed the protein level of USP11, HA-MYC, and GFP-DLGAP5 in T24 (**A**) and UM-UC-3 (**B**) cells after *USP11* knockdown and DLGAP5 overexpression. Viability of T24 (**C**) and UM-UC-3 (**D**) cells with indicated treatment after 48 h GEM treatment at various concentrations, determined using the MTT assay (n = 6). The asterisk indicates statistical significance between siNC+DLGAP5 and siU+DLGAP5. (**E**) Co-IP assays demonstrated that exogenous USP11 and MYC interactions increased with elevating DLGAP5. Statistical significance of data was ascertained by one-way ANOVA with Tukey's multiple comparisons test analyses (C, D). All statistical data are presented as mean ± SD, * $p < 0.05$, ** $p < 0.01$, *** $p < 0.001$.

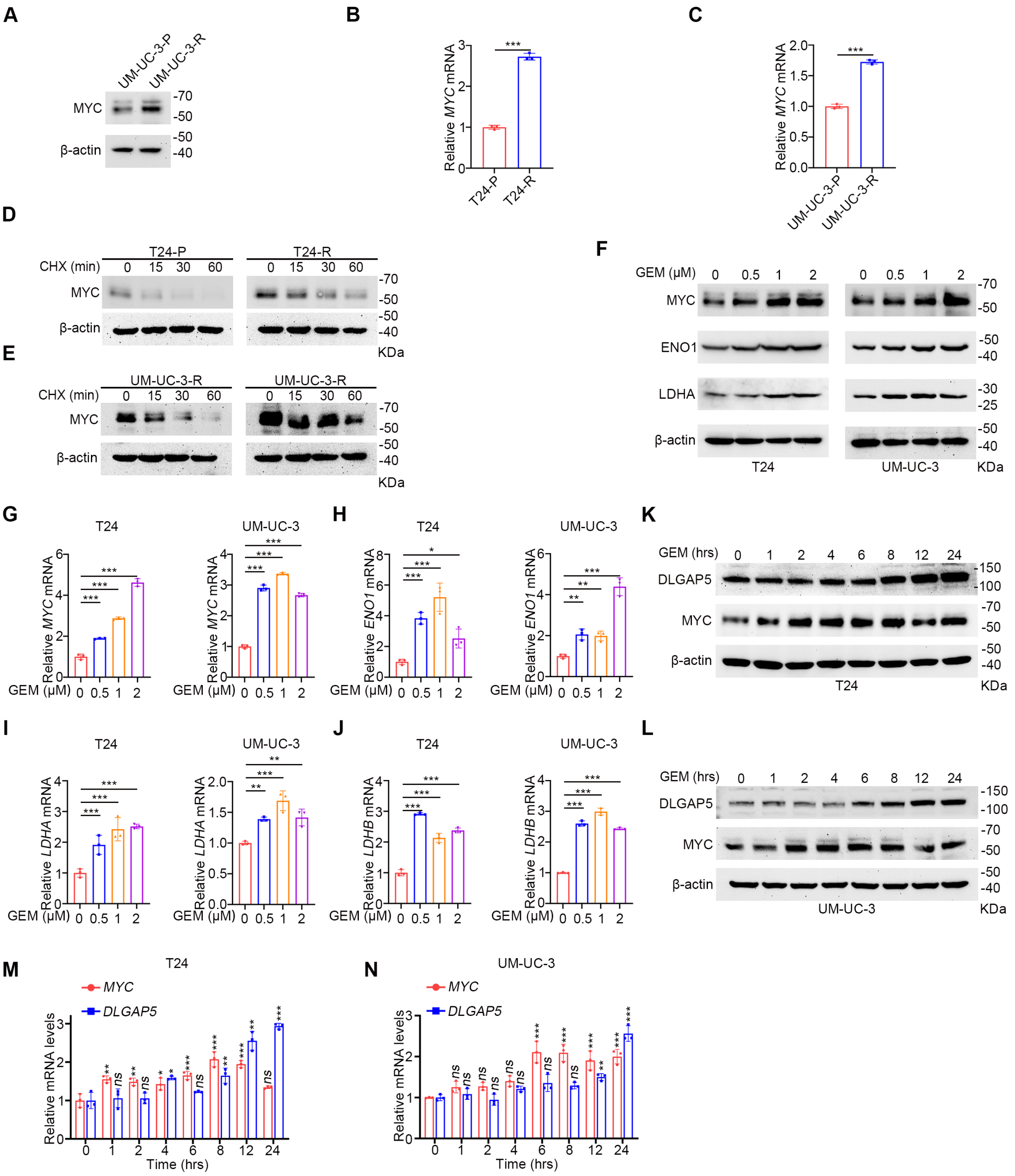

Figure S15. The DLGAP5-USP11-MYC feedback loop induces GEM resistance in BLCA cells.

(**A**) Western blot analysis of MYC proteins in UM-UC-3-P and UM-UC-3-R cells. Quantification of mRNA expression of *MYC* in T24-P and T24-R cells (**B**) and UM-UC-3-P and UM-UC-3-R cells (**C**) via qRT-PCR (n = 3). Western blot analysis of the MYC degradation in T24-P and T24-R cells (**D**) and UM-UC-3-P and UM-UC-3-R (**E**) incubated with CHX (50 μg/mL) for the indicated time points. (**F**) Western blot analysis of MYC proteins in T24 and UM-UC-3 cells after 24 h of treatment with different concentrations of GEM. Quantification of *MYC* (**G**), *ENO1* (**H**), *LDHA* (**I**), and *LDHB* (**J**) mRNA expression in T24 and UM-UC-3 cells after 24 h GEM treatment at various

concentrations via qRT-PCR (n = 3). Western blot analysis of DLGAP5 and MYC proteins in T24 (**K**) and UM-UC-3 (**L**) cells after different times of treatment with 1 μM GEM. Quantification of *MYC* and *DLGAP5* mRNA expression in T24 (**M**) and UM-UC-3 (**N**) cells after different times of treatment with 1 μM GEM (n = 3). Statistical significance of data was ascertained by two-tailed unpaired Student's t-test (B, C) and one-way ANOVA with Tukey's multiple comparisons test analyses (G-J, M, N). All statistical data are presented as mean ± SD, * $p < 0.05$, ** $p < 0.01$, *** $p < 0.001$.

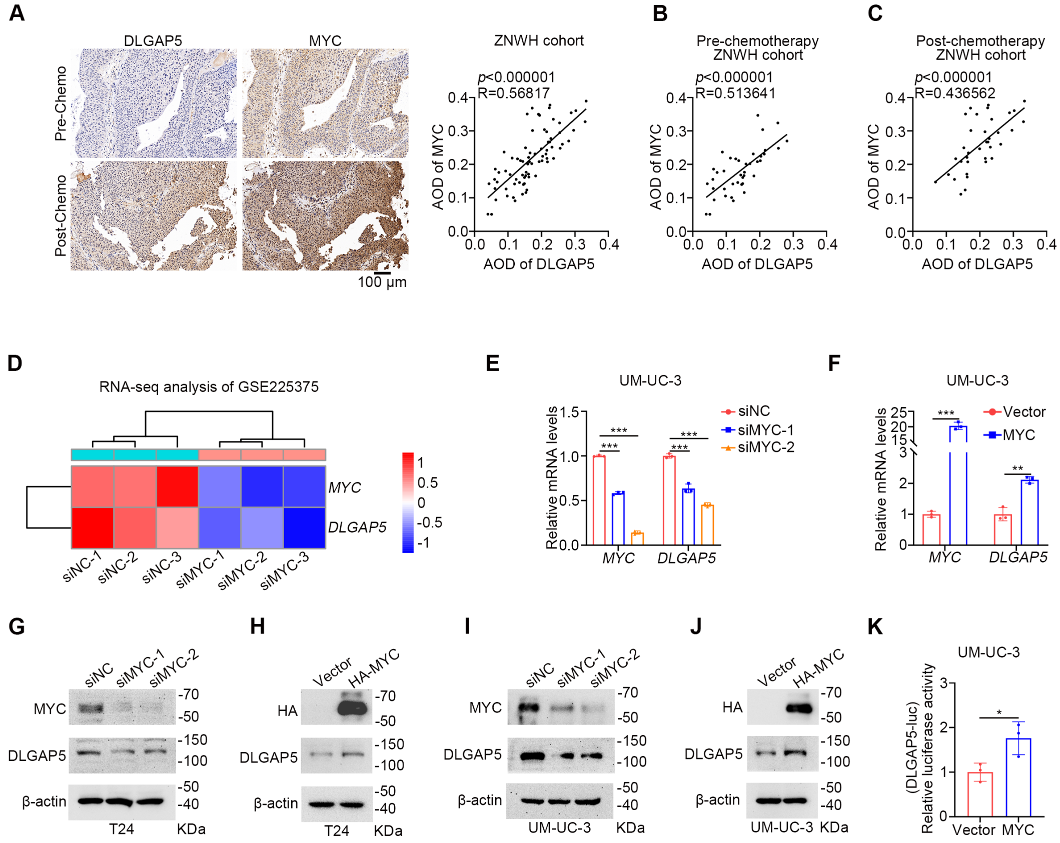

Figure S16. MYC regulates the transcription of *DLGAP5*.

(**A**) Representative images of IHC staining of DLGAP5 and MYC in human BLCA specimens from the Zhongnan Hospital of Wuhan University (ZNWH) cohort. Scale bars, 100 μm. Pearson correlation analysis was used to determine the degree of association between DLGAP5 and MYC via IHC staining (n = 77). p-value was obtained by Student's t-test. Pearson correlation analysis to determine the degree of association between DLGAP5 and MYC by IHC staining in pre-chemotherapy samples (**B**, n = 41) and post-chemotherapy samples (**C**, n = 36). p-value was obtained by Student's t-test. (**D**) A heatmap of *DLGAP5* and *MYC* upon *MYC* knockdown in high-risk Group 3 medulloblastoma cells (n = 3). UM-UC-3 cells were knocked down (**E**) and overexpressed (**F**) MYC, and mRNA levels were detected via qRT-PCR (n = 3). Western blot analysis of knockdown (**G**) and overexpressing MYC (**H**) on DLGAP5 proteins in T24 cells. Western blot analysis of knockdown (**I**) and overexpressing (**J**) MYC on DLGAP5 proteins in UM-UC-3 cells. (**K**) Dual-luciferase reporter assay of *DLGAP5* promoter activity after overexpressing MYC in UM-UC-3 cells (n = 3). Statistical significance of data was ascertained by two-tailed unpaired Student's t-test (F, K) and one-way ANOVA with Tukey's multiple comparisons test analyses (E). All statistical data are presented as mean ± SD, * $p < 0.05$, ** $p < 0.01$, *** $p < 0.001$.

Supplementary Tables

Table S1. Clinicopathological characteristics of the TCGA cohort.

		DLGAP5 expression			
	**Variables**	**High (n=45)**	**Low (n=161)**	***p*-value**	**Statistics method (two-tailed)**
**Mean age (SD)**		68.38 (9.40)	68.58 (11.30)	0.914	Chi-square
**Gender (%)**	Female	13 (28.9)	46 (28.6)	1	Chi-square
	Male	32 (71.1)	115 (71.4)		
**Stage (%)**	Unknown	1 (2.2)	0 (0.0)	0.2	Fisher's exact
	Stage I	0 (0.0)	1 (0.6)		
	Stage II	17 (37.8)	48 (29.8)		
	Stage III	15 (33.3)	50 (31.1)		
	Stage IV	12 (26.7)	62 (38.5)		
**Grade (%)**	Unknown	1 (2.2)	1 (0.6)	0.149	Fisher's exact
	High grade	44 (97.8)	150 (93.2)		
	Low grade	0 (0.0)	10 (6.2)		
**OS (%)**	0	18 (40.0)	98 (60.9)	**0.02**	Fisher's exact
	1	27 (60.0)	63 (39.1)		

DLGAP5 expression group: The optimal cut-point of the *DLGAP5* mRNA expression was cut-off value.

Statistical significance: Determined by two-tailed Chi-square or two-tailed Fisher's exact test. No adjustments were made for multiple comparisons.

Table S2. AOD of DLGAP5 and MYC for BLCA patients.

Patient number	Pre-chemotherapy		Post-chemotherapy	
	DLGAP5	MYC	DLGAP5	MYC
Patient 1	0.213	0.231	0.157	0.124
Patient 2	0.112	0.219	0.143	0.111
Patient 3	0.159	0.201	0.220	0.267
Patient 4	0.062	0.186	0.200	0.247
Patient 5	0.093	0.180	0.255	0.295
Patient 6	0.181	0.231	0.224	0.369
Patient 7	0.195	0.346	0.162	0.171
Patient 8	0.193	0.208	0.303	0.361
Patient 9	0.060	0.155	0.158	0.350
Patient 10	0.043	0.142	0.068	0.147
Patient 11	0.153	0.167	0.162	0.221
Patient 12	0.144	0.168	0.164	0.207
Patient 13	0.073	0.176	0.232	0.252
Patient 14	0.120	0.142	0.173	0.301
Patient 15	0.281	0.270	0.296	0.302
Patient 16	0.129	0.217	0.262	0.260
Patient 17	0.088	0.237	0.330	0.327
Patient 18	0.134	0.146	0.225	0.267
Patient 19	0.256	0.248	0.289	0.354
Patient 20	0.061	0.128	0.172	0.368
Patient 21	0.254	0.324	0.152	0.250
Patient 22	0.146	0.140	0.216	0.298
Patient 23	0.209	0.304	0.218	0.208
Patient 24	0.094	0.127	0.144	0.209
Patient 25	0.171	0.202	None	None
Patient 26	0.150	0.161	None	None
Patient 27	0.064	0.094	None	None
Patient 28	0.040	0.051	None	None
Patient 29	0.154	0.116	None	None
Patient 30	0.204	0.224	None	None
Patient 31	0.202	0.211	None	None
Patient 32	0.154	0.177	None	None
Patient 33	0.121	0.091	None	None
Patient 34	0.183	0.166	None	None
Patient 35	0.216	0.242	None	None
Patient 36	0.137	0.211	None	None
Patient 37	0.098	0.105	None	None
Patient 38	0.143	0.169	None	None
Patient 39	0.149	0.143	None	None
Patient 40	0.051	0.051	None	None
Patient 41	0.116	0.101	None	None

Patient number	Pre-chemotherapy		Post-chemotherapy	
	DLGAP5	MYC	DLGAP5	MYC
Patient 42	None	None	0.236	0.335
Patient 43	None	None	0.334	0.389
Patient 44	None	None	0.125	0.155
Patient 45	None	None	0.107	0.169
Patient 46	None	None	0.128	0.217
Patient 47	None	None	0.191	0.350
Patient 48	None	None	0.166	0.234
Patient 49	None	None	0.226	0.377
Patient 50	None	None	0.217	0.218
Patient 51	None	None	0.228	0.277
Patient 52	None	None	0.163	0.162
Patient 53	None	None	0.236	0.218

AOD: Average optical density.

BLCA: Bladder cancer.

ZNWH cohort_BLCA total: Patients 1-53 (n = 53).

ZNWH cohort_BLCA subgroup: Patients 1-24 (n = 24).

Table S3. Sequences of siRNAs used in this study.

siRNA	Sequences (5’ – 3’)
siDLGAP5-1	GCAAUGAGAGAGAGAAUUATT
siDLGAP5-2	GGAGCAGACUAAGAUUGAUTT
siDLGAP5-3’UTR	CUGUGUUCAUCAAAGUGUAUU
siUSP11-1	ACCGAUUCUAUUGGCCUAGUA
siUSP11-2	CUGCGUCGGGUACGUGAUGAA
siMYC-1	GCUUGUACCUGCAGGAUCUTT
siMYC-2	GGAAGAAAUCGAUGUUGUUTT
siNC	UUCUCCGAACGUGUCACGUTT

Table S4. Primers used in this study.

Assay	Gene name	Forward (5' – 3')	Reverse (5' – 3')
qRT-PCR	ALDOA	CATTCTGGCTGCGGATGAGTCT	CACACGGTCATCAGCACTGAAC
	DLGAP5	TAATGCCCACGTCGTTGAGAA	GCAGCTCTTGTGACTGGCTT
	ENO1	GTTCACAGCCAGTGCAGGAA	GGAGGCAGTTGCAGGACTTC
	GLUT1	CTTTGTGGCCTTCTTTGAAGT	CCACACAGTTGCTCCACAT
	GPI	GGAGACCATCACGAATGCAGA	TAGACAGGGCAACAAAGTGCT
	HK2	GAGCCACCACTCACCCTACT	CCAGGCATTCGGCAATGTG
	LDHA	ACGTGCATTCCCGATTCCTT	GGAAAAGGCTGCCATGTTGG
	LDHB	TGGTATGGCGTGTGCTATCAG	TTGGCGGTCACAGAATAATCTTT
	MYC	CTGGTGCTCCATGAGGAGA	CCTGCCTCTTTTCCACAGAA
	PFKP	GCATGGGTATCTACGTGGGG	CTCTGCGATGTTTGAGCCTC
	PGAM1	TTGAATACAGCGACCCAGTGG	CTATCGATGTACAGCCGAATGGTG
	PGK1	GCTCATAAGGACTACCGACTTGG	TGGACGTTAAAGGGAAGCGG
	PKM2	ATGTCGAAGCCCCATAGTGAA	TGGGTGGTGAATCAATGTCCA
	TPI1	AGTGACTAATGGGGCTTTTACTG	GCCCAATCAGCTCATCTGACTC
	USP11	TATAAGCAGTGGGAGGCATACG	ATGACCTTGCGTTCAATGGGT
	β-actin	GATCCACATCTGCTGGAAG	CAGCACAATGAAGATCAAGA
ChIP	DLGAP5-P1	TTCAGCCTCCCAAGTAGTGG	GCAAGACCCCATCTCTACCA
	DLGAP5-P2	CACACCCGGCTAATTTTTGT	GGGATCACGAGGTCAAGAGA
	DLGAP5-P3	CGCAGACCCAAAAGAGTACC	CACCACATCAGCAACCACTC
	DLGAP5-P4	CCCTGAATCCAGCTTGGTC	CCACTACGCCTGGCTAACTT
	DLGAP5-P5	GGTTTGAACACAGCAGCTCA	TCTCTGTCCCTTAGGCTGGA

qRT-PCR: Quantitative reverse transcription PCR.

ChIP: Chromatin immunoprecipitation.

Table S5. Primary antibodies used in this study.

Antibody	Source	Catalog	Dilution or amount	Host
β-actin	Proteintech	66009-1-Ig	WB/1:5000	Mouse
c-MYC	Abcam	ab32072	ChIP/1 μg	Rabbit
c-MYC	CST	18583	IP/1 μg; WB/1:1000	Rabbit
c-MYC	Proteintech	67447-1-Ig	IHC/1:500	Mouse
DLGAP5	ABclonal	A2197	WB/1:1000; IHC/1:100	Rabbit
ENO1	Abcam	ab227978	WB/1:1000	Rabbit
IgG	Proteintech	B900610	IP/1 μg	Rabbit
LDHA	CST	3582	WB/1:1000	Rabbit
USP11	ABclonal	A19562	WB/1:1000	Rabbit
Flag-tag	Sigma	F1804	IP/1 μg; WB/1:1000; IF/1:100	Mouse
Flag-tag	Proteintech	20543-1-AP	WB/1:1000	Rabbit
GFP-tag	Santa Cruz	SC-9996	IP/1 μg; WB/1:1000; IF/1:100	Mouse
GFP-tag	Proteintech	50430-2-AP	WB/1:1000	Rabbit
HA-tag	Origene	TA180128	IP/1 μg; WB/1:1000	Mouse
HA-tag	ABclonal	AE105	WB/1:1000; IF/1:100	Rabbit
Myc-tag	Abclonal	AE010	WB/1:1000	Mouse
GST-tag	Proteintech	10000-0-AP	WB/1:1000	Mouse

WB: Western blot.

ChIP: Chromatin immunoprecipitation.

IP: Immunoprecipitation.

IHC: Immunohistochemistry.

IF: Immunofluorescence.
